# A conserved *HH-Gli1-Mycn* network regulates heart regeneration from newt to human

**DOI:** 10.1038/s41467-018-06617-z

**Published:** 2018-10-12

**Authors:** Bhairab N. Singh, Naoko Koyano-Nakagawa, Wuming Gong, Ivan P. Moskowitz, Cyprian V. Weaver, Elizabeth Braunlin, Satyabrata Das, Jop H. van Berlo, Mary G. Garry, Daniel J. Garry

**Affiliations:** 1 Lillehei Heart Institute, University of Minnesota, Minneapolis, MN 55455 USA; 2 Departments of Pediatrics, Pathology, and Human Genetics, University of Chicago, Chicago, IL 60637 USA

## Abstract

The mammalian heart has a limited regenerative capacity and typically progresses to heart failure following injury. Here, we defined a hedgehog (HH)-Gli1-Mycn network for cardiomyocyte proliferation and heart regeneration from amphibians to mammals. Using a genome-wide screen, we verified that HH signaling was essential for heart regeneration in the injured newt. Next, pharmacological and genetic loss- and gain-of-function of HH signaling demonstrated the essential requirement for HH signaling in the neonatal, adolescent, and adult mouse heart regeneration, and in the proliferation of hiPSC-derived cardiomyocytes. Fate-mapping and molecular biological studies revealed that HH signaling, via a HH-Gli1-Mycn network, contributed to heart regeneration by inducing proliferation of pre-existing cardiomyocytes and not by de novo cardiomyogenesis. Further, *Mycn* mRNA transfection experiments recapitulated the effects of HH signaling and promoted adult cardiomyocyte proliferation. These studies defined an evolutionarily conserved function of HH signaling that may serve as a platform for human regenerative therapies.

## Abstract (web summary)

Due to the limited proliferation capacity of adult mammalian cardiomyocytes, the human heart has negligible regenerative capacity after injury. Here the authors show that a Hedgehog-Gli1-Mycn signaling cascade regulates cardiomyocyte proliferation and cardiac regeneration from amphibians to mammals.

## Introduction

In contrast to mammals, lower vertebrates such as the adult newt and zebrafish can achieve complete heart regeneration following injury by activating developmental regulatory networks^1–5^. In these organisms, adult cardiomyocytes undergo dedifferentiation to re-enter the cell cycle and, ultimately, differentiation to facilitate tissue regeneration^6,7^. Using these model organisms, studies have defined the activation of signaling pathways including: FGF, Notch, and BMP signals. However, little is known whether these same factors promote cardiomyocyte proliferation in mammals^8–10^. Recently, Aguirre et al. have shown that activation of a conserved microRNA pathway in the injured zebrafish heart can promote mammalian heart regeneration^11^. While these findings support the existence of conserved regenerative programs, additional studies are urgently needed to define and activate the dormant pathways in mammals.

The neonatal mammalian heart harbors a tremendous potential to promote cardiomyocyte proliferation to facilitate repair and/or regeneration^12^. In the neonatal mouse, the cardiomyocyte proliferative capacity diminishes rapidly within a 1-week period following birth^12–14^. In contrast, only limited cardiomyocyte turnover occurs in the adult mammalian heart, a capacity that is insufficient to repair or regenerate the injured heart^15,16^. Therefore, efforts have focused on the role of pathways and factors that promote cardiomyocyte proliferation and tissue regeneration in the adult mammalian heart that can prevent the progression of heart failure and premature death following cardiac injury.

Transcriptional networks and signaling pathways that govern embryonic heart development have received intense interest^13,17–22^. Importantly, these networks and pathways likely serve as a platform for cardiac regeneration following injury. Studies focused on hedgehog (HH) signaling support the critical role of this pathway during cardiovascular development in mammals^23^. Deletion of either *Smo* (*Smo*^*−/−*^) or *Ptc1* (*Ptc1*^*−/−*^), or double knockouts of *Shh;Ihh* (*Shh*^*−/−*^; *Ihh*^*−/−*^) results in embryonic lethality due to cardiovascular defects^23^. In addition, the hedgehog downstream effectors, Gli1, Gli2, and Gli3, function in a redundant and reciprocal fashion to modulate hedgehog activity in a context-dependent fashion during development. While the role of HH signaling is described in cardiac development, its role as a regulator of cardiomyocyte proliferation during heart regeneration remains unknown.

Here, we used the newt, mouse, and human heart models to discover important regulators of cardiomyocyte proliferation and regeneration. We identified a previously undefined evolutionary conserved role for HH signaling in the postnatal heart following injury. Using pharmacological inhibitors, bioinformatics, genetic gain- and loss-of-function strategies, we demonstrate a reciprocal, functional, modulatory effect on the proliferative program in cardiomyocytes. Mechanistically, we define a novel *HH-Gli1-Mycn* gene regulatory network that regulates cardiomyocyte proliferation and promotes heart regeneration.

## Results

### HH signaling is induced upon injury and is essential for heart regeneration in vivo

**Fig. 1:**
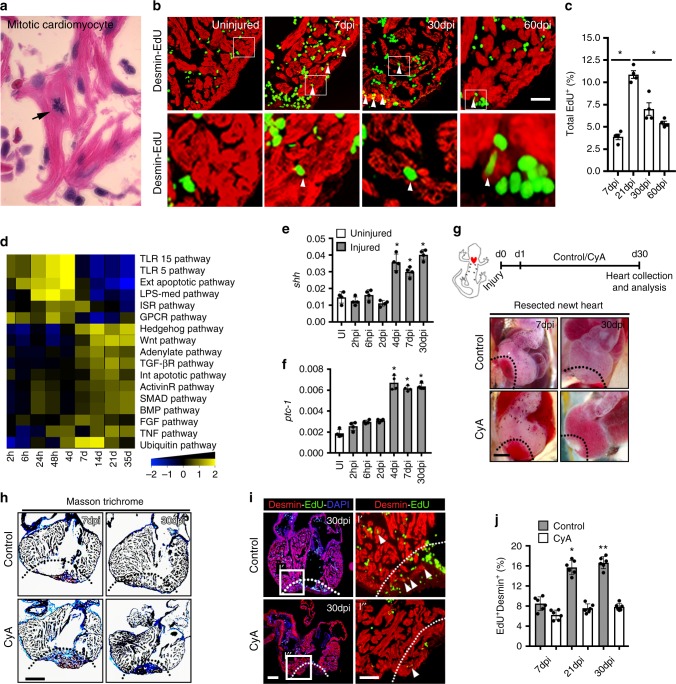
HH signaling is essential for heart regeneration. **a** Histological examination of the regenerating newt heart showing a mitotic cardiomyocyte in the injured tissue. **b**, **c** Immunohistochemical (**b**) and quantitative analysis of the total EdU^+^ cells (**c**) in the regenerating newt heart. The white arrowheads indicate the proliferating cardiomyocytes within the myocardium at specified time periods following injury. The boxed regions are magnified in the lower panels. Quantitative analysis represents counts from four randomly selected fields at 20× magnification from four replicates at each time period. **d** Gene set enrichment analysis using *Bootstrap* tools from regenerating newt heart tissue at the designated time periods postinjury. The color scale indicates the scaled expression levels of signaling pathways following heart injury. **e**, **f** qPCR analysis for *shh* and *ptc-1* transcripts during cardiac regeneration in the newt (*n* = 4). **g** Schematic (top) of experimental protocol and whole-mount images of the regenerating heart obtained from control- and CyA-treated newts at 7 dpi and 30 dpi (*n* = 8 for each group). The dotted line represents the injured region of the heart. **h** Masson Trichrome staining of the regenerating hearts from control- and CyA-treated newts at 7 dpi and 30 dpi (*n* = 8 for each group). The dotted line represents the injured region of the heart. **i**, **j** Immunohistochemical staining (**i**) and quantification (**j**) of Desmin^+^-EdU^+^ cardiomyocytes in the regenerating heart from control- and CyA-treated newts at the designated time periods following injury (*n* = 6). The dotted line in panel i represents the injured region of the heart and the tissue in the boxed region is magnified in I′ and I″ panels. White arrowheads indicate the EdU^+^-cardiomyocytes. Data are presented as mean ± SEM (*p < 0.05; **p < 0.01) (see also Supplementary Figs 1 and  2) and scale bars = 200 μm (panels **a**, **b**, **h**, **i**) and 500 μm (panel **g**). Statistical tests were done using two-tailed unpaired Student’s *t*-test and one-way ANOVA

Multiple lines of evidence support the conclusion that the adult newt harbors a tremendous regenerative capacity following cardiac injury^1,3,24^. To identify signaling networks during cardiac regeneration, we initially performed ventricular apical resection studies in the adult newt and defined its regenerative properties. Our analysis revealed complete cardiac regeneration with functional restoration of the resected (~25–30%) heart by 60 days postinjury (dpi) (Supplementary Fig. 1a–c). Our initial histological examination revealed mitotic cardiomyocytes in the regenerating newt heart tissue following apical resection injury (Fig. 1a). An EdU incorporation experiment labeled proliferating cardiomyocytes (desmin^+^-EdU^+^ cells) throughout the regenerating newt heart (Fig. 1b, c). Quantitative analysis revealed desmin^+^-EdU^+^ cardiomyocytes in the injured zone (20 ± 3%), border zone (6 ± 1%), and remote zone (2.5 ± 0.5%) of the total cardiomyocyte pool at 21 dpi (Fig. 1b, c and Supplementary Fig. 1d), suggesting a global regenerative response following resection injury.

To investigate and define the molecular signals regulating regeneration, we used the *Bootstrap* bioinformatics tool, and analyzed the microarray datasets^24^ (http://newt-omics.mpi-bn.mpg.de) from the regenerating newt heart at selected time periods following apical resection injury. Gene set enrichment analysis showed two distinct phases of response to injury. Multiple inflammatory pathways were upregulated during the early regenerative period (Fig. 1d and Supplementary Fig. 1e). By 7 dpi, inflammatory signals were downregulated with the subsequent activation of multiple signaling pathways including the hedgehog (HH) signaling pathway (Fig. 1d and Supplementary Fig. 1e). HH signals were sustained throughout the later stages of regeneration (Fig. 1d). To validate these results, we performed qPCR using RNA isolated from the regenerating heart at selected time periods. Consistent with the *Bootstrap* analysis, the gene encoding the ligand of the HH pathway, *shh*, and the HH target and co-receptor Patched, *ptc-1*, were both upregulated at 4 dpi and had sustained expression in the regenerating heart (Fig. 1e, f). Further, qPCR using RNA isolated from the bulbous arteriosus [BA (i)], atrial [AT (ii)], and ventricular [Ven (iii)] tissues, showed maximal expression of *shh* and *ptc-1* levels in the injured ventricle and BA (Supplementary Fig. 1f–h). These results supported the notion that the HH signaling pathway was important for cardiac regeneration.

We initially tested the hypothesis that HH signaling was essential for newt heart regeneration by ablating HH signals in vivo using a potent Smoothened (Smo) antagonist, cyclopamine (CyA)^25^. Continuous blockade of HH signals led to complete ablation of heart regeneration and induction of scar formation following injury (*n* = 8) (Fig. 1g, h). To further characterize the perturbed regeneration upon HH signaling inhibition, we examined cellular proliferation using an EdU-labeling assay at multiple time periods following ventricular resection. The EdU incorporation assay showed a twofold reduction in cardiomyocyte proliferation upon inhibition of HH signaling at 21 dpi and 30 dpi (*n* = 6; *p* < 0.05 and *p* < 0.01, respectively) (Fig. 1i, j). Next, we evaluated whether other lineages such as the epicardial cells were modulated by HH signaling during the regenerative period. We observed that the Wt1^+^ epicardial cell population was activated and proliferated following cardiac resection. Furthermore, the proliferation of the Wt1^+^ epicardial cell population was diminished with cyclopamine-mediated HH signaling inhibition (Supplementary Fig. 2a–c). These results showed the necessity of HH signaling for heart regeneration in the newt following injury. Importantly, our studies were further supported by others demonstrating the permissive role of signaling pathways on the epicardium and cardiovascular lineages in the regenerating zebrafish heart^26^.

### Activation of HH signaling promotes mouse neonatal cardiomyocyte proliferation in vitro

**Fig. 2:**
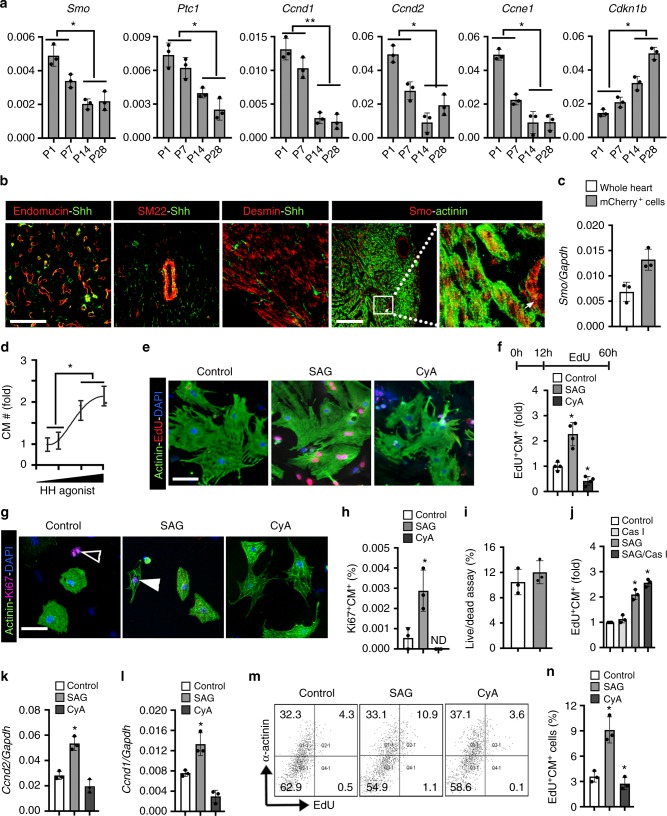
HH signaling regulates proliferation in mouse neonatal cardiomyocytes. **a** qPCR analysis of *Smo*, *Ptc1*, *Ccnd1, Ccnd2, Ccne1*, and *Cdkn1b (p27)* transcripts using RNA isolated from P1-P28 mouse heart tissue (*n* = 3 for each time point). **b** Immunostaining of Shh and Smo proteins with Endomucin (endothelial), SM22 (smooth muscle), Desmin (cardiomyocytes), and α-Actinin (cardiomyocytes) in P1 mouse heart sections. The boxed region is magnified in the right panel. The white arrow indicates the staining of Smo in the vascular structure. Note the punctate staining of Smo in the cardiomyocytes. **c** qPCR analysis for *Smoothened* transcripts using RNA isolated from whole heart and FACS-sorted αMHC-mCherry^+^ cells (a transgenic cardiomyocyte-specific promoter driving mCherry expression) from P1–P2 pooled hearts. **d** Quantitative analysis of cultured neonatal cardiomyocytes following treatment with various concentration of SAG. **e**, **f** Immunohistochemical images (**e**) and quantification (**f**) of α-Actinin^+^-EdU^+^ isolated neonatal cardiomyocytes following exposure to control (white bar), SAG (gray bar), or CyA (dark gray bar) and pulsed with EdU. Quantitative analysis represents the counting of four different fields at 10× from four replicates (*n* = 2000 cardiomyocytes for each condition). **g**, **h** Immunohistochemical images (**g**) and quantification (**h**) of α-Actinin^+^-Ki67^+^ isolated neonatal cardiomyocytes following exposure to control (white bar), SAG (gray bar), or CyA (dark gray bar). Quantitative analysis represents the counting of three different fields at 10× from three replicates (*n* = 2000 cardiomyocytes for each condition). Open arrowhead indicate non-cardiomyocytes and closed arrowhead cardiomyocyte positive for Ki67 protein. **i** Live/Dead assay using the isolated neonatal cardiomyocytes following exposure to control (white bar) and SAG (gray bar). Quantitative analysis represents the counting of three different fields at 10× from three replicates. **j** Quantification of α-Actinin^+^-EdU^+^ isolated neonatal cardiomyocytes following exposure to DMSO (Control), pan-caspase inhibitor (Cas I), SAG and (SAG+Cas I) for a 48 h period. Quantitative analysis represents the counting of eight different fields at 10× from three replicates. **k**, **l** qPCR analysis for *Ccnd2* and *Ccnd1* transcripts from isolated neonatal cardiomyocytes exposed to DMSO, SAG or CyA. **m**, **n** FACS analysis (**m**) and quantification (**n**) for α-Actinin^+^-EdU^+^ cardiomyocytes in control (white bar), SAG (gray bars) and CyA (dark gray bars) treated conditions. Quantification involved the analysis of cardiomyocytes (*n* = 30,000) from three replicates. Data are presented as mean ± SEM (*p < 0.05; **p < 0.01) (see also Supplementary Fig. 3) and scale bars = 100 μm. Statistical tests were done using two-tailed unpaired Student’s *t*-test

During mouse embryonic development, HH signaling coordinates cardiac progenitor proliferation, specification and coronary vascular development^23,27^. This ligand-receptor pathway includes hedgehog ligands (Shh, Ihh, Dhh) and membrane receptors [Smoothened (Smo) and Patched1 (Ptc1)] to regulate the downstream effectors^4,28^. While global deletion of *Smo* as well as *Shh*^*−/−*^*/Ihh*^*−/−*^ results in embryonic lethality due to cardiovascular defects^23^, its role in the postnatal proliferative myocardium and the perinatal regenerative period is unknown. To analyze the expression of HH signaling in the postnatal heart, we performed qPCR using RNA isolated from P1-P28 mouse hearts. qPCR analysis using P1 ventricular tissue revealed robust expression of the HH pathway transcripts, *Smo* and *Ptc1*, and cell cycle transcripts, *Ccnd1*, *Ccnd2*, and *Ccne1*. All of these transcripts were subsequently downregulated by P28 (Fig. 2a). In contrast, the cell cycle repressor gene, *Cdkn1b*, was upregulated between the P7-P28 period compared to P1 (Fig. 2a). The reduction in *Smo*, *Ptc1*, *Ccnd1*, *Ccnd2*, and *Ccne1* transcripts during the first week of postnatal development indicated concomitant downregulation of HH signaling and the proliferative program as the heart loses its regenerative potential.

To assess the role of HH signaling in the regenerating neonatal mouse heart, we performed immunohistochemical analysis of Shh and Smo in postnatal day 1 (P1) heart tissue sections. Shh was strongly expressed in the non-myocyte cellular pool, including the endothelium (Shh^+^-Endomucin^+^ cells) and smooth muscle cells (Shh^+^-SM22^+^ cells) (Fig. 2b). Furthermore, immunohistochemial analysis of Shh and Desmin demonstrated an absence of co-labeled cells, suggesting that Shh was not expressed in cardiomyocytes (Fig. 2b). In contrast, the immunohistochemical analysis of Smo and Actinin revealed punctate expression of Smo in Actinin-positive cardiomyocytes (Smo^+^-Actinin^+^ cells) as well as strong expression in the non-myocyte cellular (Smo^+^-Actinin^−^ cells; white arrow) pool (Fig. 2b). Our analysis showed an uniform staining of Smo in cardiomyocytes. To further verify the expression of *Smo* in the cardiomyocytes, we undertook qPCR analysis using FACS-sorted αMHC-mCherry^+^ cells (a transgenic cardiomyocyte-specific promoter driving mCherry expression) from P1 hearts. qPCR analysis revealed a robust expression of the *Smo* transcripts in these mCherry^+^ cells (Fig. 2c). Based on these results, we hypothesized that a Shh morphogen secreted by the non-myocyte cellular pool signaled, in a paracrine manner, the adjacent Smo-expressing cardiomyocytes.

We directly tested HH signaling activity using small molecule-mediated activation and inhibition studies on isolated mouse neonatal cardiomyocytes. In vitro administration of the HH agonist (SAG) resulted in a dose-dependent increase in the number of cultured neonatal cardiomyocytes (Fig. 2d and Supplementary Fig. 3a). We then performed an EdU incorporation assay to monitor the proliferation indices of the cultured neonatal cardiomyocytes. Compared to the controls, SAG treatment resulted in a 2.5-fold (*n* = 4; *p* < 0.05) increase in α-Actinin^+^-EdU^+^ cells. In contrast, cyclopamine (CyA)-mediated inhibition of HH signaling resulted in a significant decrease in cardiomyocyte proliferation (*n* = 4; *p* < 0.05) (Fig. 2e, f). To validate these EdU incorporation results, we performed immunostaining for Ki67 to examine the proliferating cardiomyocytes following treatment with DMSO, SAG, and CyA. Very few cardiomyocytes were stained for Ki67 (Ki67^+^-Actinin^+^ cells) in the controls, whereas, we observed an increased number of Ki67^+^-Actinin^+^ cells in the SAG-treated (*n* = 3; *p* < 0.05) cardiomyocytes. In contrast, we did not detect any Ki67^+^-Actinin^+^ cells in the CyA-treated groups (Fig. 2g, h). To evaluate whether HH signaling has a protective function, we performed a live/dead assay using the cultured cardiomyocytes following treatment with DMSO and SAG for 48 h. We did not find any difference in the live/dead assay between control- and SAG-treated cardiomyocytes (*n* = 3) (Fig. 2i). These data indicated that activation of HH signaling did not induce an anti-apoptotic pathway in the cultured cardiomyocytes. Next, to investigate a pro-proliferative impact of HH signaling, we treated the cultured cardiomyocytes with SAG in the presence or absence of a pan-caspase inhibitor and performed an EdU-incoporation assay. Pan-caspase-mediated inhibition of apoptosis in the presence of SAG did not result in any change in the EdU^+^ cardiomyocytes compared to SAG alone (*n* = 3; *p* < 0.05) (Fig. 2j). These results demonstrated a pro-proliferative impact of HH signaling in the cardiomyocytes. Next, qPCR analysis for *Ptc1*, *Ccnd2*, *Ccnd1*, and *Ccne1* transcripts confirmed the induction of cell cycle kinetics upon HH signaling activation (Fig. 2k, l and Supplementary Fig. 3b, c). Further, fluorescence-activated cell sorting (FACS) analysis of the α-Actinin^+^-EdU^+^ population demonstrated increased cardiomyocyte proliferation (2.5-fold) upon SAG treatment (*n* = 3; *p* < 0.05) (Fig. 2m, n). The increased cardiomyocyte proliferation upon SAG treatment was also evident in the serum-free conditions (*n* = 3; *p* < 0.05) (Supplementary Fig. 3d, e). These results showed that activation of HH signaling promoted the proliferation of postnatal neonatal cardiomyocytes in vitro.

### In vivo activation of HH signaling extends the cardiac regenerative window

**Fig. 3:**
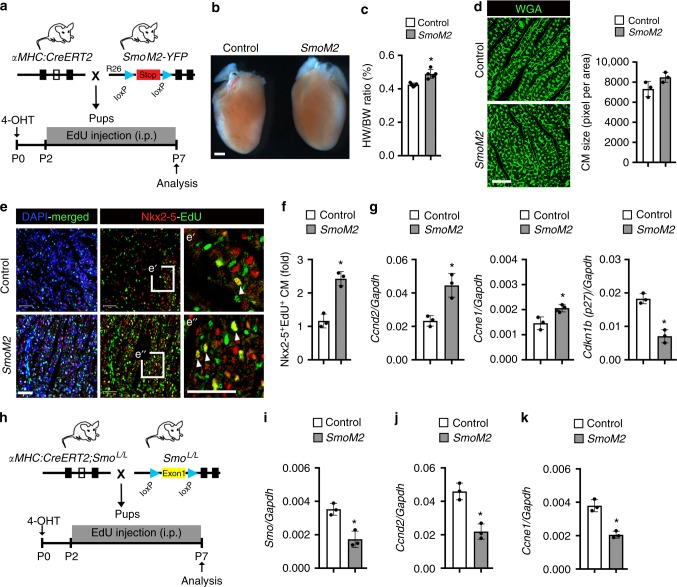
In vivo activation of HH signaling promotes cardiomyocyte proliferation. **a** Schematic for the activation of HH signaling and EdU labeling in the postnatal heart during the regenerative window (P2-P7) in the mouse. **b** Whole-mount images of representative control and *SmoM2* hearts obtained by crossing *αMHC:CreERT2* with *Rosa26-SmoM2/YFP* mice. Control mice were not injected with 4-hydroxytamoxifen. **c** Heart weight to body weight (HW/BW) ratio in control and *SmoM2* mice (*n* = 5 per group). **d** WGA staining and quantification of the heart sections from control and *SmoM2* mice at P7 (*n* = 3). **e**, **f** Immunostained images (**e**) and quantification (**f**) of Nkx2-5^+^-EdU^+^ cardiomyocytes in control and *SmoM2* hearts. The boxed regions are magnified in e′ and e″ panels. Arrowheads indicate EdU^+^-cardiomyocytes. Quantitative analysis in panel F represents the counting of four random fields at 20× magnification from three biological replicates. **g** qPCR analysis of *Ccnd2*, *Ccne1*, and *Cdkn1b* (*p27*) transcripts using RNA isolated from control and *SmoM2* heart tissue (*n* = 3) at P7. **h** Schematic outlining the experimental protocol of cardiomyocyte-specific conditional deletion of the *Smo-floxed* allele (*Smo CKO*) by crossing *αMHC:CreERT2;Smo*^*L/L*^ with *Smo*^*L/L*^ mice. **i**–**k** qPCR analysis of *Smo, Ccnd2,* and *Ccne1* transcripts using RNA isolated from control (white bar) and *Smo CKO* (gray bar) heart tissues (*n* = 3 hearts in each group). Data are presented as mean ± SEM (*p < 0.05) (see also Supplementary Fig. 4) and scale bars = 100 μm (panels **d**, **e**) and 500 μm (panel **b**) as indicated. Statistical tests were done using two-tailed unpaired Student’s *t*-test

We next investigated whether activation of HH signals could modulate the cardiomyocyte proliferative potential in vivo. To evaluate the role of HH signaling in vivo, we conditionally activated HH signaling in cardiomyocytes by crossing mouse models with a floxed allele of an active, *Smo mutant* (*SmoM2*)^29^ with a cardiomyocyte-specific tamoxifen-inducible Cre (*αMHC-CreERT2* [*αMHC-MerCreMer*])^30^. Subcutaneous injection of 4-hydroxytamoxifen (TM) in *αMHC:CreERT2;Rosa26-ZsGreen* neonates at P0/P1 resulted in specific and efficient (>95%) labeling of cardiomyocytes (Supplementary Fig. 4a, b). Hearts from TM-treated *αMHC:CreERT2;SmoM2-YFP*^*fl/+*^ (*SmoM2*) mice demonstrated increased levels of *Ptc1*, *Gli1*, and *Gli2* transcripts following TM treatment by qPCR, confirming the activation of the HH signaling cascade (Fig. 3a and Supplementary Fig. 4c–e).

Increased HH signaling in TM-treated *αMHC:CreERT2;SmoM2-YFP*^*fl/+*^ (*SmoM2*) mice revealed cardiac enlargement with increased ventricular wall thickness, heart weight to body weight (HW/BW) ratio (*n* = 5; *p* < 0.05), and heart weight to tibia length (HW/TL) ratio (*n* = 5; *p* < 0.05) (Fig. 3b, c, and Supplementary Fig. 4f, g). To determine whether cardiac enlargement was due to a hyperplastic or hypertrophic effect, we analyzed cardiomyocyte proliferation between P2-P7 (regenerative period)^12^. EdU incorporation assays revealed an increased percentage of EdU^+^ cells (*n* = 3; *p* < 0.05) and Nkx2-5^+^-EdU^+^ cardiomyocytes (*n* = 3; *p* < 0.05) in the *SmoM2* hearts without any detectable change in cardiomyocyte size relative to control hearts (Fig. 3d–f, and Supplementary Fig. 4h, i). qPCR analysis demonstrated increased levels of *Ccnd2* and *Ccne1* with reduced expression of *Cdkn1b (p27)* in *SmoM2* hearts (Fig. 3g). These results support the notion that the activation of HH signaling promotes cardiomyocyte proliferation. To further examine this hypothesis, we conditionally deleted *Smo* in the cardiomyocyte by crossing the *αMHC:CreERT2;Smo*^*L/L*^ with *Smo-floxed* (*Smo*^*L/L*^) mice at P0. Deletion of *Smo* in the cardiomyocyte resulted in reduced levels of *Ccnd2* and *Ccne1* at P7 (Fig. 3h–k). Overall, these results established that HH signaling regulated the proliferation program of the neonatal cardiomyocyte population in vivo.

**Fig. 4:**
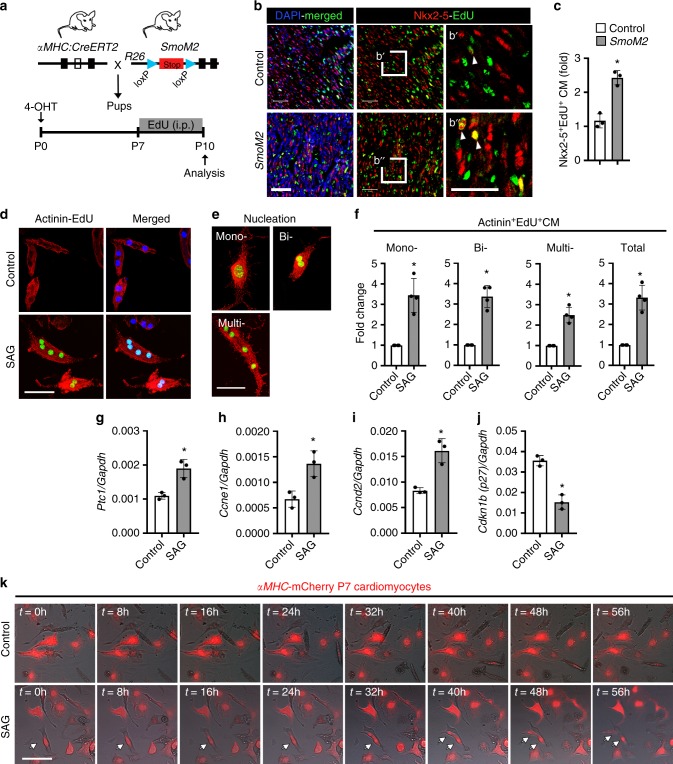
Activation of HH signaling promotes cardiomyocyte proliferation during the non-regenerative period. **a** Schematic for the activation of HH signaling and EdU labeling in the postnatal heart during the non-regenerative window (P7-P10). **b**, **c** Immunostaining (**b**) and quantification (**c**) of Nkx2-5^+^-EdU^+^ cardiomyocytes in control and *SmoM2* hearts. The boxed region in panel **b** is magnified in **b**′ and **b**″ panels. Arrowheads indicate EdU^+^ cardiomyocytes. Quantitative analysis in panel **c** represents the counting of four random fields at 20× magnification from three biological replicates. **d**–**f** Immunohistochemical images (**d**, **e**) and quantification (**f**) of α-Actinin^+^-EdU^+^ cultured P7 cardiomyocytes from control- and SAG-treated conditions. Quantitative analysis represents the counting of three random fields from four replicates (*n* = 2100 cardiomyocytes for each condition). Representative images and quantification of the number of mono-, bi-, multi-nucleated and total myocytes from control- and SAG-treated samples are shown. **g**–**j** qPCR analysis of *Ptc1*, *Ccnd2*, *Ccne1,* and *Cdkn1b (p27)* transcripts using RNA isolated from control- and SAG-treated P7 cardiomyocytes (*n* = 3). **k** Time-lapse microscopic images of αMHC-mCherry^+^ cardiomyocytes from control- and SAG-treated conditions at the specified time intervals. The white arrow indicates the dividing cardiomyocyte. Data are presented as mean ± SEM (*n* = 3; *p < 0.05) (see also Supplementary Fig. 5) and scale bars = 100 μm. Statistical tests were done using two-tailed unpaired Student’s *t*-test

Having established the role of HH signaling in the regenerative period or window (<P7)^12^, we tested whether HH signaling activation was able to promote cardiomyocyte proliferation in the non-proliferative/non-regenerative window (>P7). We pulsed *SmoM2* pups with EdU between P7-P10 (Fig. 4a). As expected, few proliferating cardiomyocytes were noted at P10 in the wild-type control. In contrast, activation of Smo (*SmoM2*) resulted in an increased percentage of Nkx2-5^+^-EdU^+^ cells (approximately twofold) relative to control (*n* = 3; *p* < 0.05) (Fig. 4b, c). To confirm these in vivo findings, we isolated P7 cardiomyocytes (>95% rod-shaped binucleated cardiomyocytes), treated them with SAG and examined the cardiomyocyte proliferation. Remarkably, SAG treatment resulted in an increased number of α-Actinin^+^-EdU^+^ cardiomyocytes with a significant increase (approximately threefold) in the number of mono-, bi-, and multi-nucleated cardiomyocytes (*n* = 4; *p* < 0.05) (Fig. 4d–f). SAG treatment of P7-isolated cardiomyocytes resulted in increased expression of *Ptc1*, *Ccne1*, and *Ccnd2*, and reduced expression of *Cdkn1b (p27)* as detected by qPCR (Fig. 4g–j). To further monitor the HH signaling-mediated activation of cardiomyocyte cell division, we undertook time-lapse microscopic experiments using αMHC-mCherry^+^ cardiomyocytes isolated from P7 mice following treatment with either DMSO (control) or SAG. It is important to note that P7 cardiomyocytes have a relatively low proliferative ability as compared to P1–P2 cardiomyocytes. Similar to the immunostaining results, we could not detect dividing P7 cardiomyocytes in the control conditions (Fig. 4k; Supplementary Movie 1). Interestingly, we observed dividing αMHC-mCherry^+^ P7 cardiomyocytes in the SAG treatment condition (Fig. 4k; Supplementary Movie 2). Overall, these findings clearly demonstrated a proliferative role of HH signaling in the postnatal cardiomyocytes. We next investigated the ability of HH signaling to activate proliferation during the late juvenile stage (P28) (Supplementary Fig. 5a). We observed a significant increase in heart size (*n* = 4; *p* < 0.05) (Supplementary Fig. 5b–d), in the number of EdU^+^ cells (twofold), and in the number of Nkx2-5^+^-EdU^+^ cardiomyocytes (1.8-fold) (*n* = 3; *p* < 0.05) in TM-treated *SmoM2* mice (Supplementary Fig. 5e, f). Collectively, these results demonstrated that HH signaling activation was sufficient to extend cardiomyocyte proliferation during the non-proliferative/non-regenerative window.

### HH signaling regulates mammalian heart regeneration following injury

**Fig. 5:**
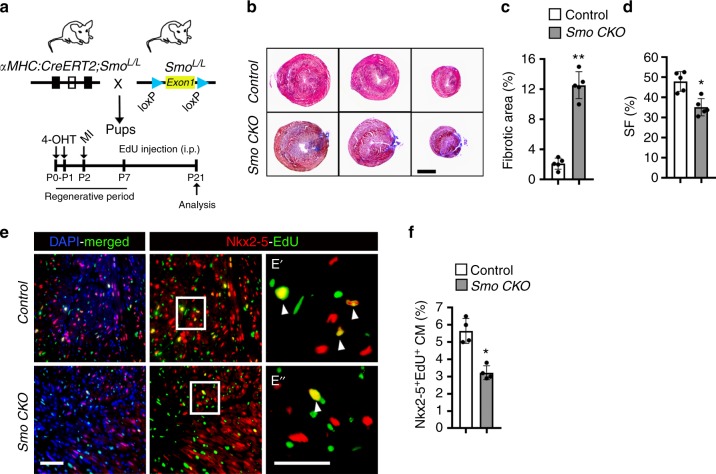
Conditional deletion of *Smoothened* (*Smo CKO*) results in impaired neonatal heart regeneration following myocardial injury. **a** Schematic of cardiomyocyte-specific conditional deletion of the floxed-Smo allele and heart regeneration analysis following MI. The MI was performed on P2. **b**, **c** Masson trichrome staining of representative sections (B) and fibrotic area quantification (**c**) of heart sections at 21 days postinjury (*n* = 5 for each group). **d** Echocardiographic measurements (SF) of cardiac function from control and *Smo CKO* at 21 days post-MI (*n* = 5 per group). **e**, **f** Immunostaining (**e**) and quantification (**f**) of Nkx2-5^+^-EdU^+^ cardiomyocytes from control and *Smo CKO* at 21 days post-MI (*n* = 4 per group). The boxed region in the middle panel of “**e**” is magnified in panels E′ and E″. Arrowheads indicate EdU^+^ cardiomyocytes. Quantitative analysis represents counting of three random fields at 20× magnification from four biological replicates in each group. Data are presented as mean ± SEM (*p < 0.05, **p < 0.01) and scale bars = 100 μm. Statistical tests were done using two-tailed unpaired Student’s *t*-test

Having established that HH signals could modulate cardiomyocyte proliferation both in vitro and in vivo, we directly evaluated the role of HH signaling during heart regeneration following injury. To determine whether HH signaling is required for heart regeneration during the regenerative window (<P7), we tested whether conditional deletion of floxed-*Smo* (*Smo*^*L/L*^) resulted in impaired neonatal heart regeneration. We injected 4-hydroxytamoxifen (TM) in the *αMHC:CreERT2;Smo*^*L/L*^ (*Smo CKO*) neonatal pups at P0 and P1, and performed myocardial infarction (MI) injury by ligating the left anterior descending (LAD) coronary artery at P2 (regenerative period) (Fig. 5a). Previous studies have shown a robust regenerative potential in the P2 heart following injury^12^. Regeneration in TM-treated *Smo CKO* and control mice was analyzed at P21 following MI (Fig. 5a). While the control hearts regenerated normally, *Smo CKO* hearts showed impaired regeneration and scar tissue formation at 21 days post-MI (*n* = 5; *p* < 0.01) (Fig. 5b, c). To further validate these results, we undertook a functional analysis of the control and *Smo CKO* regenerating hearts. Echocardiographic data analysis revealed decreased cardiac function at 21 days post-MI (*n* = 5; *p* < 0.05) (Fig. 5d). Next, we performed an EdU-labeling assay using the regenerating tissues from control and *Smo CKO* hearts. *Smo CKO* mice showed a reduced percentage of Nkx2-5^+^-EdU^+^ cardiomyocytes relative to controls (*n* = 4; *p* < 0.05) using immunohistochemical analysis (Fig. 5e, f). These results indicated that HH signaling is required for cardiomyocyte proliferation and heart regeneration in vivo.

**Fig. 6:**
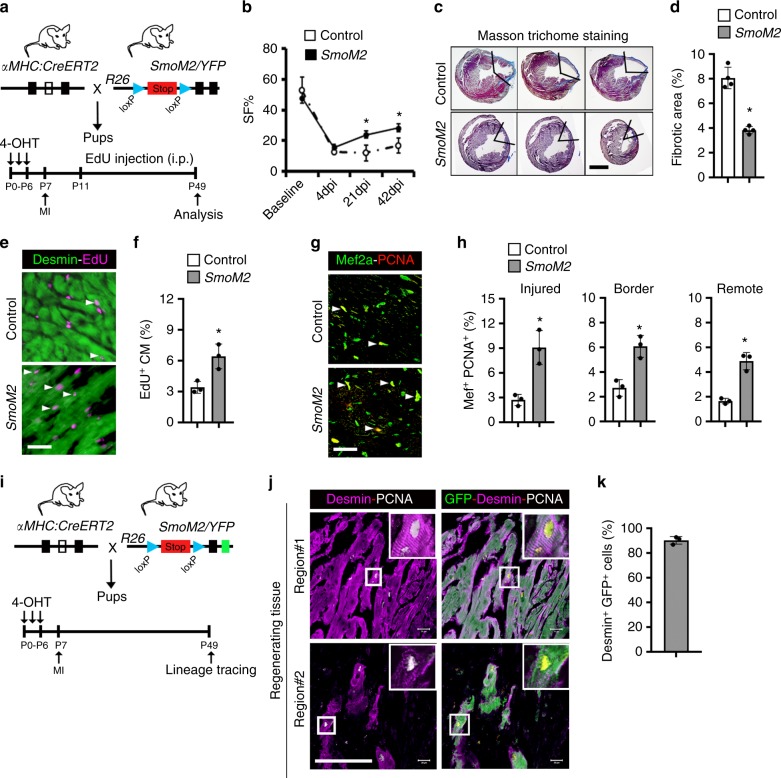
Activated *Smoothened* (*SmoM2*) augments heart regeneration by promoting proliferation of pre-existing cardiomyocytes during the non-regenerative period. **a** Schematic outlining the experimental design for Smo activation (SmoM2) and cardiac regeneration analysis following myocardial injury. **b** Time series echocardiographic measurement of shortening fraction (SF%) of control and *SmoM2* mice following MI (*n* = 4 per group). **c**, **d** Masson trichrome staining (**c**) and fibrotic area quantification (**d**) of representative heart sections at 42 days postinjury from control and *SmoM2* hearts (*n* = 4 for each group). **e**, **f** Immunostaining (**e**) and quantification (**f**) of Desmin^+^-EdU^+^ cardiomyocytes in control and *SmoM2* hearts. Arrowheads indicate EdU^+^ labeled cardiomyocytes. **g**, **h** Immunostaining (**g**) and quantification (**h**) of Mef2a^+^-PCNA^+^ cardiomyocytes in injured, border, and remote areas from control and *SmoM2* hearts (*n* = 3 for each group). Arrowheads indicate PCNA^+^-cardiomyocytes. Quantitative analysis represents counting three random fields at 20× magnification from three biological replicates. **i** Schematic outlining the lineage tracing experiment to examine the impact of the activation of HH signaling on the pre-existing cardiomyocytes following myocardial injury. **j**, **k** Immunostaining (**j**) and quantification (**k**) of Desmin^+^-PCNA^+^-GFP^+^ cardiomyocytes in control and *SmoM2* hearts at 42 days post-MI. The boxed region is further magnified and shown in the right corner of the images. Data are presented as mean ± SEM (*p < 0.05) (see also Supplementary Fig. 6) and scale bars = 100 μm (panel **c**) and 200 μm (panels **e**, **g**, **j**). Statistical tests were done using two-tailed unpaired Student’s *t*-test and one-way ANOVA

Based on these results, we next hypothesized that HH signaling activation may promote the regenerative response in vivo during the non-regenerative period (>P7). We examined the effect of HH signaling activation following MI injury by ligating the LAD coronary artery at P7 (Fig. 6a). Activating HH signaling in TM-treated *SmoM2* mice led to a significant improvement in cardiac function following MI compared to controls at 21 dpi and 42 dpi (*n* = 4; *p* < 0.05) (Fig. 6b). We found extensive scarring and loss of myocardial tissue in the control hearts. In contrast, TM-treated *SmoM2* hearts revealed cardiac regeneration with a significantly reduced fibrotic area (*n* = 4; *p* < 0.05) (Fig. 6c, d). Immunohistochemical analysis of the regenerating tissue was performed to visualize the cellular proliferation upon HH activation. *SmoM2*-expressing hearts revealed increased Desmin^+^-EdU^+^ cardiomyocytes (2.1-fold; *n* = 3; *p* < 0.05) with a higher percentage of Desmin^+^-PCNA^+^ cardiomyocytes (fourfold; *n* = 3; *p* < 0.05) and Desmin^+^-pH3^+^ (twofold; *n* = 3; *p* < 0.05) cardiomyocytes compared to controls (Fig. 6e, f, and Supplementary Fig. 6a–d). Furthermore, we observed a significantly higher percentage of Mef2a^+^-PCNA^+^ cardiomyocytes (greater than twofold; *n* = 3; *p* < 0.05) in the injured, border, and remote areas of the *SmoM2*-expressing hearts relative to controls (Fig. 6g, h). Next, lineage tracing experiments were performed to monitor the contribution of pre-existing cardiomyocytes to the regenerating tissue (Fig. 6i). These experiments demonstrated that *SmoM2*-expressing hearts induced the proliferative response in pre-existing cardiomyocytes, and that largely (>80%) contributed to the regenerating heart (Fig. 6j, k). These results indicated that HH signaling was able to extend the temporal window for heart regeneration in vivo.

**Fig. 7:**
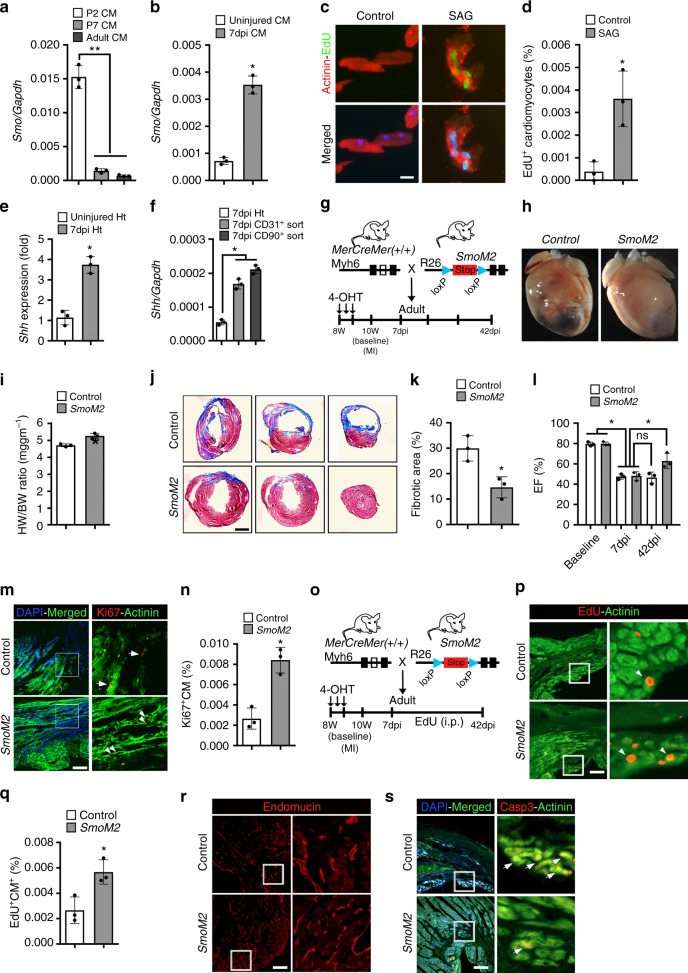
Constitutively active *Smoothened* mutant (*SmoM2*) promotes adult heart regeneration. **a** qPCR analysis of *Smoothened* (*Smo*) transcripts using RNA obtained from isolated cardiomyocytes at P2, P7, and P60 mouse hearts (*n* = 3 for each time period). **b** qPCR analysis of *Smoothened* (*Smo*) transcripts using RNA obtained from isolated cardiomyocytes from adult uninjured and injured heart tissue at 7 dpi (*n* = 3 for each sample). **c**, **d** Immunohistochemical images (**c**) and quantification (**d**) of α-Actinin^+^-EdU^+^ isolated adult cardiomyocytes following exposure to control (white bar) and SAG (gray bar) and pulsed with EdU. Quantitative analysis represents the counting of four different fields at 10× from three replicates (*n* = 1500 cardiomyocytes for each condition). **e** qPCR analysis of *Shh* transcripts using RNA obtained from adult uninjured and injured heart tissue at 7 dpi (*n* = 3). **f** qPCR analysis of *Shh* transcripts using RNA obtained from adult injured heart tissue, FACS-sorted CD31^+^ and CD90^+^ cells at 7 days post-MI (*n* = 3). **g** Schematic outlining the experimental design for *Smo* activation (*SmoM2*) and cardiac regeneration analysis following myocardial injury. **h** Whole-mount images of representative control and *SmoM2* hearts following MI. Control mice were injected with corn oil. **i** Heart weight to body weight (HW/BW) ratio in control and *SmoM2* adult mice at 42 dpi (*n* = 3 for each group). **j**, **k** Masson trichrome staining (**j**) and fibrotic area quantification (**k**) of representative heart sections at 42 days postinjury from control and *SmoM2* hearts. **l** Time series echocardiographic measurement of ejection fraction (EF%) of control and *SmoM2* mice following MI (*n* = 3 per group). **m**, **n** Immunostaining (**m**) and quantification (**n**) of α-Actinin^+^-Ki67^+^ cardiomyocytes in control and *SmoM2* hearts. Arrowheads indicate Ki67^+^ labeled cardiomyocytes and arrows indicate Ki67^+^ labeled non-cardiomyocytes. Quantitative analysis represents counting three different fields at 20× magnification. The boxed region is magnified and shown in the right panel. **o** Schematic outlining the EdU-pulse experiment and cardiac regeneration analysis following myocardial injury. **p**, **q** Immunostaining (**p**) and quantification (**q**) of Actinin^+^-EdU^+^ cardiomyocytes in control and *SmoM2* hearts. Arrowheads indicate EdU labeled cardiomyocytes. Quantitative analysis represents counting three different fields at 20× magnification near the injured area. The boxed region is magnified and shown in the right panel. **r** Immunostaining of control and *SmoM2*-expressing heart tissue sections using endomucin antibodies at 42 dpi. The boxed region is magnified and shown in the right panel. **s** Immunostaining of control and *SmoM2*-expressing heart tissue sections using α-Actinin and cleaved caspase-3 antibodies at 42 dpi. The white arrows indicate α-Actinin^+^-caspase-3^+^ cardiomyocytes. The boxed region is magnified and shown in the right panel. Data are presented as mean ± SEM (*p < 0.05) and scale bars = 100 μm. Statistical tests were done using two-tailed unpaired Student’s *t*-test and one-way ANOVA

To determine the regulatory role of Smo during adult mouse heart regeneration, we examined *Smoothened* (*Smo*) transcript expression using RNA obtained from the isolated cardiomyocytes at P2, P7, and P60 stages and performed qPCR analysis. We found robust expression of *Smo* in the P2 FACS-sorted αMHC-mCherry^+^ cardiomyocytes (Fig. 7a). The level of *Smo* transcripts were decreased at subsequent stages with limited expression in isolated P60 cardiomyocytes (Fig. 7a). To examine the expression of *Smo* transcripts following injury, we performed LAD coronary artery ligation injury in the adult mouse heart and isolated cardiomyocytes at 7 dpi following injury. qPCR analysis of the freshly isolated adult cardiomyocytes at 7 dpi revealed increased expression of *Smo* in the injured cardiomyocytes relative to uninjured cardiomyocytes (Fig. 7b). To monitor whether activated *Smoothened* could modulate regeneration capacity of the adult mouse heart, we isolated adult mouse cardiomyocytes, treated them with SAG, and performed EdU incorporation assays. We observed no or minimal α-Actinin^+^-EdU^+^ cardiomyocytes in the control or untreated adult cardiomyocytes. Notably, SAG-mediated activation of HH signaling led to an increased number of α-Actinin^+^-EdU^+^ cardiomyocytes (*n* = 3; *p* < 0.05) (Fig. 7c, d). These results demonstrated that HH signaling activation was sufficient to induce proliferation in the mature cardiomyocytes. Next, we evaluated the role of HH signaling during adult heart regeneration following injury. Initially, we investigated the expression of *Shh* transcripts following myocardial injury (LAD ligation). qPCR analysis revealed a fourfold increase in the levels of *Shh* transcripts in the injured heart tissue relative to uninjured tissue (Fig. 7e). To evaluate the source of Shh transcripts, we sorted CD31^+^ cells (endothelial lineage) and CD90^+^ cells (fibroblast population) from the injured heart tissue and performed qPCR experiments at 7 dpi. Our analysis revealed robust expression of Shh in both CD31^+^ and CD90^+^ lineages, indicating that these cells could be the major source of Shh morphogen (Fig. 7f). To interrogate the functional role of HH signaling during following injury in the adult heart, we used adult *αMHC:CreERT2;SmoM2-YFP*^*fl/+*^ (*SmoM2*) mice, and activated HH signaling in cardiomyocytes by subcutaneous injection of 4-hydroxytamoxifen (TM) at P56 (8 weeks (8 W) old). Following corn oil/TM-injection, the animals were allowed to recover for 1 week and MI injury was performed at 10 W of age (Fig. 7g). Whole-mount image analysis revealed an enlarged heart with increased heart weight to body weight ratio in the TM-treated *SmoM2* mice following injury at 42 dpi (Fig. 7h, i). Subsequent analysis of the whole-mount images demonstrated the presence of scar tissue in the control animals, whereas TM-treated *SmoM2* mice showed relatively less scarring (Fig. 7h). Further, histological examination showed extensive scarring and loss of myocardial tissue in the control hearts, correlating with the lack of regeneration in the adult tissue (Fig. 7j). Remarkably, TM-treated *SmoM2* hearts revealed significantly reduced fibrotic area following ischemic injury (*n* = 3; p < 0.05) (Fig. 7j, k). Importantly, activation of HH signaling in TM-treated *SmoM2* mice led to a significant improvement in ejection fraction (EF) as compared to controls at 42 dpi (*n* = 3; *p* < 0.05) (Fig. 7l). To determine whether the decreased fibrosis in the TM-treated *SmoM2* hearts was due to increased cardiomyocyte proliferation, we undertook immunohistochemical analysis using Ki67-antibodies. We observed an increased number of Ki67^+^-Actinin^+^ cells in the *SmoM2* hearts as compared to control tissue (*n* = 3; p < 0.05) (Fig. 7m, n). To further validate these results, we pulsed the LAD-ligated animals with EdU (i.p.) every 3 days post-MI and performed an EdU incorporation assay at 42 dpi following injury (Fig. 7o). TM-treated *SmoM2*-expressing hearts revealed an increase in the number of Actinin^+^-EdU^+^ cardiomyocytes (1.8-fold; *n* = 3; p < 0.05) compared to controls (Fig. 7p, q). These experiments demonstrated that *SmoM2*-expressing hearts induced the proliferative response following injury in the adult animals. Previous reports have suggested that Shh may have a global anti-apoptotic as well as neovascularization roles in the injured heart^31^. To determine whether cardiomyocyte-specific *SmoM2* expression is associated with increased neovascularization following MI, we performed endomucin staining at 42 days post-MI. Our immunofluorescent analysis showed a mild increase in the vascular structures in the *SmoM2*-expressing hearts as compared to control hearts (Fig. 7r). We next performed cleaved caspase-3 immunostaining and found low numbers of α-Actinin^+^-caspase-3^+^ cardiomyocytes (*n* = 3) in the *SmoM2*-expressing hearts as compared to control hearts (Fig. 7s). These results provide compelling evidence for the essential role of HH signaling during heart regeneration in adult animals.

### Conserved role of HH signaling in the regulation of cardiomyocyte proliferation

Having described the role of HH signaling in the regulation of cardiomyocyte proliferation and regeneration in both newt and mouse (Figs. 1–7), we examined the conserved function of HH signaling using human-induced pluripotent stem cell (hiPSC)-derived cardiomyocytes (hiPSC-CMs). We differentiated hiPSCs using a protocol that yields functional cardiomyocytes^32^. Using this protocol, we obtained robustly beating cardiomyocytes with high efficiency (>78% cTnT+cardiomyocytes) at day 60 (Supplementary Fig. 7a, b). We examined the effect of increased HH signaling on the proliferation of terminally differentiated hiPSC-CMs (Supplementary Fig. 7c–f). Administration of SAG to day 60 hiPSC-CMs resulted in increased α-Actinin^+^-EdU^+^ cardiomyocytes (twofold; *n* = 3; *p* < 0.01) and α-Actinin^+^-Ki67^+^ cardiomyocytes (approximately threefold; *n* = 3; *p* < 0.01), respectively (Supplementary Fig. 7c–f). In contrast, CyA-treatment resulted in a significant decrease (*n* = 3; *p* < 0.05) in the proliferation of day 60 hiPSC-CMs (Supplementary Fig. 7c–f). These findings support an evolutionary conserved role for HH signaling in the regulation of cardiomyocyte proliferation.

### Gli1-Mycn cascade regulates the cardiomyocyte proliferative response

**Fig. 8:**
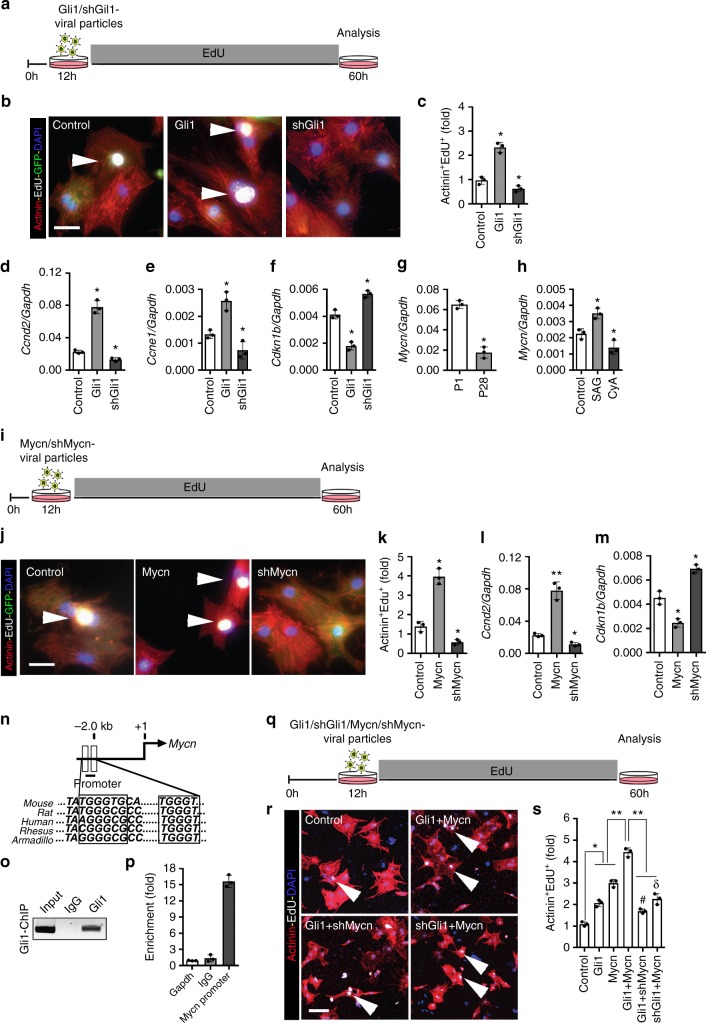
*HH-Gli1-Mycn* network regulates cardiomyocyte proliferation. **a** Schematic of *Gli1* overexpression and knockdown experiments in the neonatal cardiomyocytes. **b**, **c** Immunostaining (**b**) and quantification of α-Actinin^+^-EdU^+^ cells (**c**) from control, Gli1, and shGli1 lentiviral infected cardiomyocytes. Quantitative analysis represents counting from four randomly selected fields at 10× magnification from three biological replicates. **d**–**f** qPCR analysis of *Ccnd2*, *Ccne1*, and *Cdkn1b (p27)* following lentiviral Gli1 overexpression or knockdown (shGli1) in the P1 cardiomyocytes. **g** qPCR analysis of *Mycn* transcripts using RNA isolated from P1 and P28 wild-type heart tissue (*n* = 3). **h** qPCR analysis of *Mycn* transcripts using RNA isolated from control, SAG, and CyA treated isolated neonatal cardiomyocytes (*n* = 3 replicates from each group). **i** Schematic of Mycn overexpression and knockdown experiments in the P1 cardiomyocytes. **j**, **k** Immunostaining (**j**) and quantification of α-Actinin^+^-EdU^+^ cells (**k**) from control, Mycn, and shMycn lentiviral infected neonatal cardiomyocytes. Quantification was performed from three biological replicates. **l**, **m** qPCR analysis of *Ccnd2* and *Cdkn1b (p27)* in the cultured cardiomyocytes following Mycn overexpression and knockdown (shMycn) conditions (*n* = 3 for each group). **n** Schematic showing the Mycn genomic locus (top panel) harboring evolutionary conserved Gli1 binding motifs. **o** ChIP-PCR and quantification (**p**) for the *Mycn* promoter region following immunoprecipitation for endogenous Gli1 using isolated neonatal cardiomyocytes. **q** Schematic of combinatorial lentiviral infection studies using *Gli1*, *shGli1*, *Mycn,* and *shMycn* viral particles. **r**, **s** Immunostaining (**r**) and quantification of α-Actinin^+^-EdU^+^ cells (**s**) from control, *Gli1*, *shGli1*, *Mycn,* and *shMycn* (using Clone A; see Supplementary Fig. 8j) infected using isolated neonatal cardiomyocytes. Quantitative analysis represents counting of three random fields from three replicates (*n* = 1000 cardiomyocytes for each condition). Arrowheads indicate EdU^+^ labeled cardiomyocytes Data are presented as mean ± SEM (*p < 0.05; **p < 0.01; ^#^represents significance (*p* < 0.05) between Gli1+shMycn compared Gli1 conditions; ^δ^represents significance (*p* < 0.05) between shGli1+Mycn compared to Mycn conditions) (see also Supplementary Fig. 8) and scale bars = 200 μm. Statistical tests were done using two-tailed unpaired Student’s *t*-test and one-way ANOVA

To decipher the mechanism by which HH signaling mediates cardiomyocyte proliferation, we analyzed the expression of the downstream effectors *Gli1* and *Gli3* from the P1-P28 mouse heart. We observed robust Gli1 expression at P1, which was essentially extinguished by P28; conversely, *Gli3* expression was low at P1 and increased significantly by P28 (Supplementary Fig. 8a, b). Activation of HH signaling by SAG treatment induced *Gli1* and reduced *Gli3* expression. Conversely, CyA-treatment caused reduced *Gli1* and increased *Gli3* expression in isolated mouse neonatal cardiomyocytes (Supplementary Fig. 8c, d). To further explore Gli1-dependent regulation of cardiomyocyte proliferation, we performed lentiviral-mediated overexpression and knockdown of Gli1 in neonatal cardiomyocytes (Fig. 8a and Supplementary Fig. 8e, f). Overexpression of Gli1 by lentiviral particles resulted in increased α-Actinin^+^-EdU^+^ cardiomyocytes (twofold; *n* = 3; *p* < 0.05) with higher levels of *Ccnd2* and *Ccne1* transcripts (Fig. 8b–e). In contrast, Gli1-knockdown (shGli1) led to impaired cardiomyocyte proliferation (*n* = 3; *p* < 0.05) and reduced expression of cyclins with a concomitant increase in *Cdkn1b (p27)* levels (Fig. 8b–f).

Next, to identify downstream targets of Gli1, we used Gli1-ChIPseq data sets^33^ and examined putative candidates based on their expression in the cardiomyocyte, ChIPseq binding proximity, and proliferative function. Using these criteria, we prioritized the candidates and identified Mycn as the top-ranked candidate (Supplementary Table 1). Notably, *Mycn* transcripts paralleled *Gli1* expression with increased expression in the P1 heart and extinguished expression by P28 (Fig. 8g). The *Mycn* transcripts were increased following SAG treatment, whereas CyA-treatment resulted in reduced expression in isolated mouse neonatal cardiomyocytes (Fig. 8h). Lentiviral-mediated *Gli1* overexpression or knockdown in isolated neonatal cardiomyocytes led to an increase or decrease in *Mycn* transcripts, respectively (Supplementary Fig. 8g, h), suggesting that the function of Gli1 was mediated through Mycn.

**Fig. 9:**
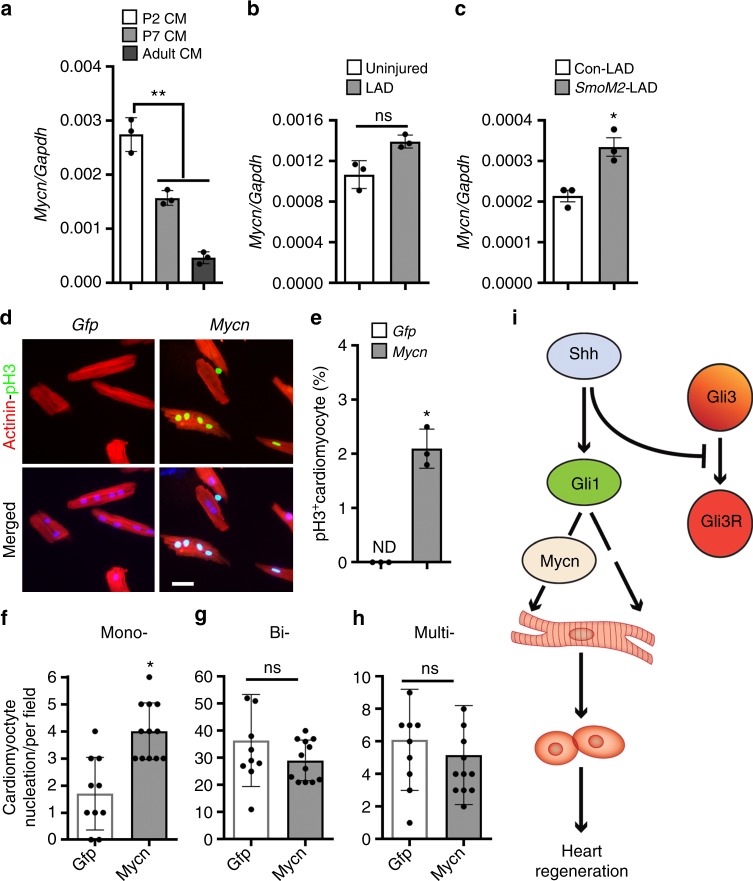
*Mycn* recapitulates HH signaling-mediated adult cardiomyocyte proliferation. **a** qPCR analysis of *Mycn* transcripts using RNA obtained from isolated cardiomyocytes at P2, P7, and P60 mouse hearts (*n* = 3). **b** qPCR analysis of *Mycn* transcripts using RNA obtained from adult uninjured and injured heart tissue at 7 days post-MI. **c** qPCR analysis of *Mycn* transcripts using RNA obtained from control and *SmoM2*-expressing heart tissue following 7 days post-MI. **d**, **e** Immunohistochemical images (**d**) and quantification (**e**) of α-Actinin^+^-pH3^+^ isolated adult cardiomyocytes following transfection with *Gfp* and *Mycn mRNAs* at 48 h. Quantitative analysis represents the counting of ten different fields at 10× from three replicates (*n* = 450 cardiomyocytes for each condition). **f**–**h** Quantitative analysis of the number of mono-, bi-, and multi-nucleated cardiomyocytes from *Gfp* and *Mycn mRNAs* transfected adult cardiomyocytes. Quantitative analysis represents the counting from multiple fields at 10× magnification from three replicates. **i** Schematic model depicting the Shh-Gli1-Mycn regulatory network and cardiomyocyte proliferation. Gli1 transcription factor is induced as a downstream effector of HH signaling upon the binding of the Shh morphogen to its membrane receptor. Gli1 and Gli3 function in an antagonistic fashion as Gli1 promotes proliferation whereas Gli3 acts to repress the proliferative program and induces maturation. Activated Gli1 transactivates its downstream target, Mycn, to regulate the proliferative response in cardiomyocytes. Data are presented as mean ± SEM (*p < 0.05; **p < 0.01). Scale bars = 200 μm. Statistical tests were done using two-tailed unpaired Student’s *t*-test

We examined the direct effect of Mycn on neonatal cardiomyocyte proliferation using lentiviral-mediated overexpression and knockdown of Mycn (Fig. 8j and Supplementary Fig. 8i, j). Induction of Mycn led to a significant increase in the number of α-Actinin^+^-EdU^+^ cardiomyocytes (3.5-fold; *n* = 3; *p* < 0.05) with increased *Ccnd2* transcript expression (Fig. 8j–l). Conversely, the knockdown of Mycn resulted in a decreased proliferative response and increased *Cdkn1b (p27)* levels in the cardiomyocytes (Fig. 8j–m). To examine Gli1–Mycn interaction in vivo, we performed chromatin immunoprecipitation (ChIP)-PCR for endogenous Gli1 proteins using isolated neonatal cardiomyocytes and demonstrated Gli1 binding to the *Mycn* promoter (Fig. 8n–p). To further define the *Gli1-Mycn* regulatory pathway, we performed combinatorial lentiviral infection studies using *Gli1*, *shGli1, Mycn*, and *shMycn* viral particles in isolated neonatal murine cardiomyocytes (Fig. 8q). Co-expression of Gli1 and Mycn resulted in a robust increase (fivefold; *n* = 3; *p* < 0.01) in the EdU^+^-cardiomyocytes, suggesting an additive role of these two factors (Fig. 8r, s). Importantly, induction of Mycn together with Gli1-knockdown or *vice-versa* resulted in impaired proliferative index in isolated cardiomyocytes as compared to Gli1 or Mycn by itself (Fig. 8r, s). To determine the proliferative role of Mycn in the adult mouse myocardium, we examined the expression analysis of *Mycn* transcripts using isolated cardiomyocytes from three postnatal stages (P2, P7, and P60). Our qPCR analysis revealed robust expression of *Mycn* in the P2 FACS-sorted αMHC-mCherry^+^ cardiomyocytes (*p* < 0.01) (Fig. 9a). Low levels of *Mycn* transcripts were detected at subsequent stages with limited expression in the P60 isolated cardiomyocytes (Fig. 9a). Next, we examined the expression of *Mycn* transcripts following LAD coronary artery ligation injury in the adult heart at 7 dpi. qPCR analysis at 7 dpi showed a nonsignificant change in the levels of *Mycn* in the injured hearts as compared to uninjured tissue (*n* = 3) (Fig. 9b). To evaluate whether the levels of *Mycn* transcripts were altered following activation of HH signaling, we performed LAD ligation injury in control- and TM-treated *SmoM2*-expressing hearts and analyzed its expression at 7 dpi. qPCR analysis revealed a significant increase in the *Mycn* transcripts in the *SmoM2*-expressing hearts as compared to control injured hearts (*n* = 3; *p* < 0.05) (Fig. 9c). These results further supported the notion that HH signaling is upstream of the Mycn regulatory network in vivo. To monitor whether overexpression of Mycn could recapitulate the impact of HH signaling as a stimulator of adult cardiomyocyte proliferation (Fig. 7c, d), we undertook *Gfp* and *Mycn mRNA* transfection experiments and performed the proliferative assay. The number of transfected GFP^+^ cells did not change until 48 h post-transfection (Supplementary Fig. 9a, b). We examined cardiomyocyte proliferation using the mitosis marker pH3 (phosphorylated histone H3) and α-Actinin antibodies. We were unable to detect any pH3^+^-Actinin^+^ cells in the *Gfp* transfected controls, indicating low or no proliferative capacity of the adult mature cardiomyocytes. *mRNA*-mediated overexpression of *Mycn* resulted in a significant induction of cardiomyocyte proliferation as measured by the increased percentage of pH3^+^-Actinin^+^ cells relative to the *Gfp* transfected cells (*n* = 3 experiments; *p* < 0.05) (Fig. 9d, e). Remarkably, *Mycn* transfections led to a significant increase (~1.9-fold) in the number of mono-nucleated cardiomyocytes, with no significant changes in the bi- and multi-nucleated myocytes (*n* = 3; *p* < 0.05) (Fig. 9f–h). These results clearly support the notion that Mycn is one of the downstream effectors of Gli1 in cardiomyocyte proliferation and regeneration both in vitro as well as in vivo (Fig. 9i).

## Discussion

Evolutionary conserved mechanisms that govern development and cellular proliferation have been well described^4,11,13,34,35^. These studies have uncovered important mechanisms in lower organisms such as the fly, zebrafish, frogs, and newt, and have been used to interrogate and modulate pathways in mammalian organs^1–3,11,24,36,37^. These strategies have led to discoveries including Hippo signaling, Wnt signaling, Notch signaling, and other pathways that regulate tissue regeneration in lower organisms, and have been shown to harbor a similar role in mammalian organisms^38,39^. Several signaling factors, transcription factors, and microRNAs have been shown to regulate cardiogenesis, however, their roles in the postnatal heart following injury are unclear. In the present study, we used the power of the regenerating newt heart, genomics, and pharmacological perturbations, as well as genetic perturbations to show the impact of HH signaling on cardiomyocyte proliferation from newt to mouse to human. We also made three important discoveries.

First, we defined the role of hedgehog signaling as a promoter of cardiomyocyte proliferation. Moreover, our studies demonstrated a pro-proliferative effect of HH signaling without impacting the apoptotic pathway. The hedgehog signaling pathway has been shown to have essential roles during embryogenesis^29,36^. Previous studies from our laboratories and others have identified a role for the HH signaling pathway in regulating progenitor cell proliferation and angiogenesis^4,5,40^. Global deletion of the ligand, Sonic hedgehog, and/or its G protein-coupled receptor, Smoothened (Smo), resulted in early embryonic lethality and perturbed cardiogenesis in the mouse^23^. Similarly, gene disruption studies of the HH downstream effectors (Gli1/Gli2/Gli3) demonstrated perturbed cardiogenesis^41^. Previous studies have shown that the role and expression of the Gli transcription factors are context dependent^42,43^. These studies support the notion that Gli1 and Gli2 have overlapping functions as transcriptional activators, whereas Gli3 functions, in a context-dependent fashion, to repress HH signaling. Our studies support the hypothesis that HH/Gli1-dependent developmental mechanisms that govern embryogenesis are also operational during the regenerative process and are important mechanistic drivers for the regeneration of postnatal tissues. Therefore, we have expanded our understanding of the role of Gli1 from its role during development to its role in cardiac repair.

Our second discovery defined the role of Mycn as a downstream target of Gli1. Mycn is a proto-oncogene that encodes a protein that has a basic helix-loop-helix DNA-binding domain^44^. Mycn mutations are associated with Feingold syndrome, which is a disorder associated with congenital heart defects^45^. Global as well as cardiac-specific deletion of *Mycn* locus results in lethality by midgestational age and displays growth retardation and perturbed cardiogenesis^46,47^. While there are functional redundancies associated with Myc family members, the overall homology between Mycn and c-Myc is only about 30%, suggesting that specific domains harbor critical functions for these proteins for the growth and development of specific lineages^48^. In the present study, we defined Mycn as a direct downstream target of Gli1. Moreover, we defined the impact of a hedgehog-Gli1-Mycn cascade as an inducer of cardiomyocyte proliferation and a facilitator of heart regeneration following injury.

Our third discovery emphasized the essential nature of evolutionary conserved signaling pathways that can be deciphered using emerging bioinformatics algorithms, which can then be coupled with pharmacological and genetic technologies in mammalian organisms. In the present study, we used the *Bootstrap* algorithm to interrogate cardiac regeneration in the adult newt. This strategy was used to identify candidate factors/pathways that were induced and had sustained expression during cardiac regeneration. While this bioinformatics strategy successfully identified the hedgehog signaling pathway, other signaling pathways were also identified and warrant further examination. The present studies also used the neonatal mouse heart, hiPSC-derived cardiomyocytes, and genetic mouse models, which are powerful models to examine the impact of evolutionary conserved factors and their impact on cardiomyocyte proliferation. The neonatal mouse heart has a tremendous regenerative capacity which is rapidly extinguished by P7 following birth^12^. This regenerative model may serve as an extension of the developmental programs expressed during embryogenesis and is a powerful model to define regulators that promote cardiomyocyte proliferation. We took advantage of the regenerative and non-regenerative windows/periods during the postnatal heart development to interrogate and demonstrate the impact of hedgehog signaling and cardiomyocyte proliferation. Previous reports have described additional roles for HH signaling in the regulation of neovascularization and/or anti-apoptotic process following myocardial injury^31,49^. Based on our study as well as others, it is possible that HH signaling has multiple roles including proliferation, vasculogenesis, and protective functions following injury. Our data indicated that Shh was expressed and secreted as a morphogen by both CD90^+^- and CD31^+^-cell populations following injury. Therefore, we propose that Shh morphogen may function in an autocrine as well as paracrine manner, however, overexpression of Shh alone in these cells may not be sufficient to drive the pathway for effective repair. In the present study, we have provided a new mechanistic, proliferative pathway mediated via Mycn. Since the levels of both Smoothened and Mycn were low in the adult myocardium, gene therapy and/or mRNA-mediated overexpression of these factors could promote adult heart regeneration following injury.

In summary, our studies support the power of using multiple organisms to uncover evolutionary conserved networks that impact cardiomyocyte proliferation and regeneration^1–3^. Our studies also emphasize the importance of examining essential development pathways that are reexpressed following injury and function to promote regeneration. Moreover, we uncovered a novel *HH-Gli1-Mycn* regulatory mechanism that facilitates cardiomyocyte proliferation and enhances our understanding of just one of the keys that unlock the myocardial regeneration program. The highly conserved nature of these newly discovered mechanisms suggest the importance of this pathway in promoting cardiac regeneration. Successful induction of this molecular pathway holds unique potential for induction of cardiac regeneration following injury in humans.

## Methods

All animal handling and experimental procedures were approved by the Institutional Animal Care and Use Committee of the University of Minnesota. Ethical approval was obtained and adherence to ethical guidelines related to the animal studies were followed. Adult newt heart resection injury studies were performed using male and female newts. All the mouse-related experiments were performed using male mice. All experiments were repeated at least three times and the data represent the mean ± SEM.

### Newt husbandry and heart resection surgery

All experiments were performed according to the University of Minnesota IACUC guidelines. Adult red-spotted newts, *Notophthalmus viridescens*, were housed as previously described^5^. For heart resection surgery, adult newts were anesthetized in 0.1% MS-222 solution for 10 min. Each newt was placed in a supine position under a stereomicroscope. The outer skin was wiped using 70% ethanol-chlorhexidine solution and the pericardial sac was opened to expose the heart. The apex of the ventricle was resected (~25–30%) using iridectomy scissors. Following resection, the blood flow was controlled by the formation of the blood clot. The resected heart was maneuvered carefully into the pericardial sac and sutured using 8.0 ethilon monofilament sutures. Following resection surgery, newts were allowed to recover in an isolated tank containing sulfmerazine antibiotic solution and later placed in their designated aquariums. The resected ventricular mass was measured using a Sartorius weighing balance. Cyclopamine (CyA; LC laboratories) was dissolved in 100% ethanol to a stock concentration of 10 mg/ml. CyA-mediated inhibition of HH signaling was achieved by daily treatment of the resected newts at 2 µg/ml diluted in the aquarium water. At specified time periods, animals were sacrificed and tissues were collected for further processing. For EdU labeling, the resected newts were injected intraperitoneally (i.p.) with 100 μg/gm of EdU for a 7 day period prior to sacrifice and harvested for immunohistochemical analysis.

### Echocardiography

Newts were anesthetized using a 0.1% MS-222 solution and echocardiograms were obtained by placing the probe adjacent to the pericardial sac using a Vevo2100 echocardiographic machine. Diastolic and systolic dimensions were measured in a blinded fashion and the average values were used to calculate the fractional shortening at each time point. Echocardiographic analyses were performed using multiple newts for each time period.

### Histology and immunohistochemistry

For histological analysis, animals were euthanized at specified time periods and tissues were fixed in 4% paraformaldehyde. Histological sectioning, hematoxylin-eosin (H&E) staining and Masson Trichome staining were performed as previously described^5^. Immunohistochemistry was performed on cryosections (10 µm thick) using standard procedures^5,50–52^. Briefly, sections were rehydrated, permeabilized, and blocked with 10% normal donkey serum (NDS), 0.1% Triton X-100 in PBS at room temperature and incubated overnight at 4 °C with primary antibodies: α-actinin (Abcam; 1:300), desmin (Novus biologicals; 1:300), Shh (Santa Cruz Biotechnology; 1:200), cleaved caspse-3 (Cell Signaling; 1:300), endomucin (Abcam; 1:100), SM22 (Abcam; 1:400), α-phospho-Histone H3 (Ser10) (Millipore; 1:100), Ki67 (Abcam; 1:200), PCNA (Santa Cruz Biotechnology; 1:100), Mef2a (Santa Cruz Biotechnology; 1:100), Smoothened (Abcam; 1:200), Nkx 2–5 (Santa Cruz Biotechnology; 1:100), and GFP (ThermoFisher Scientific; 1:300) sera. Sections were rinsed and incubated with combinations of secondary antibodies (1:400) including Alexa 488, Alexa 594, Cy3, and Cy5 (Jackson ImmunoResearch Laboratories). EdU staining was performed using the EdU labeling kit (Life Technologies).

### Genetic mouse models

All experiments were performed according to the University of Minnesota IACUC guidelines. To activate HH signaling in a cardiomyocyte-specific fashion, we used the *αMHC:CreERT2* (*MerCreMer*) mouse strain^30^ and crossed the mice with *Rosa26-SmoM2-YFP*^*fl/+*^ mice^29^. Injection of 4-hydroxytamoxifen led to constitutive expression of the *Smo/EYFP* fusion gene and increased HH signaling in the Cre-expressing tissues. To delete Smoothened (*Smo CKO*), we crossed the *αMHC:CreERT2;Smo*^*L/L*^ mouse model with the *Smo-floxed* (*Smo*^*L/L*^) mouse lines and subcutaneously injected 4-hydroxytamoxifen (80 µg/gm) in neonates at P0/P1 stage. For the late juvenile stage, we injected 4-hydroxytamoxifen in neonates at P0, P3, and P6. Control and *SmoM2-YFP*^*fl/+*^ mice received intraperitoneal (i.p.) injections of EdU (25 µg/g) daily until P7. For the late juvenile stage, EdU injections were delivered every 3 days from P11 to P28. Heart tissues were excised following perfusion using 30 mM KCl solution followed by phosphate-buffer saline perfusion. Excised heart tissues were immersion-fixed in 4% paraformaldehyde overnight at 4 °C, and rinsed in cold PBS and processed for cryosectioning.

### MI and echocardiography

MI in neonatal (P2), juvenile (P7), and adult (P66) mice were performed by ligation of the LAD coronary artery as previously described^53,54^. Neonates and P7 mice were anaesthetized by cooling on an ice bed for 1–2 min. Lateral thoracotomy at the fourth intercostal space was achieved by blunt dissection of the intercostal muscles following skin incision. A tapered needle (C-1) attached to a 6–0 prolene suture (Ethicon) was passed through the midventricle below the origin of the LAD coronary artery and ligated to induce MI. Following ligation, thoracic wall incisions were sutured with 6.0 non-absorbable silk sutures, and the skin wound was closed. Pups were then warmed under a heat lamp for several minutes until recovery and injected with buprenorphine-SR (i.m.). Adult LAD ligation experiments were performed as described previously^54^. Briefly, hair was removed from the surgical site and disinfected with 70% isopropyl alcohol and povidone iodine solution. Adult mice were anesthetized with inhaled 2–5% isofluorane and intubated to the level of the carina and an adequate level of anesthesia was maintained using a Harvard ventilator. Using sterile procedures, a thoracotomy was performed to expose the heart and the proximal LAD coronary artery was permanently ligated below the middle region of the heart to obtain moderate injury using 6–0 silk sutures. The thoracic wall was closed using 3–0 silk sutures and the mice were extubated. After LAD ligation injury, mice were maintained on a heating platform (37 °C) and continuously monitored until they were fully recovered and ambulating about the cage. The hearts were collected for analysis at the designated end points. For echocardiography, conscious mice were restrained in a supine position and echocardiograms were obtained by placing the probe adjacent to the pericardial sac using a Vevo2100 machine.

### RNA isolation and qPCR

RNA isolation and qPCR analysis from newt tissue was performed as previously described^5^. For chamber based qPCR analysis, the regenerating heart including BA, AT, and ventricle was harvested and rinsed in PBS to remove blood cells. Subsequently, the BA, AT, and ventricle were collected from heart tissue (*n* = 12) for further processing. RNA isolation from cultured cardiomyocytes was performed using a standard protocol as per the manufacturer’s instructions. Total RNA was isolated using the miRVANA kit (Ambion) and cDNA was made using SuperScript Reverse Strand Synthesis-III kit (Invitrogen).

### Wheat germ agglutinin (WGA) staining

Cryosections were rinsed 3 times in PBS and incubated with a primary antibody against WGA conjugated to Alexa Fluor 488 (50 μg/ml, Invitrogen, CA) for 1 h at room temperature. Slides were then rinsed in PBS and DAPI staining was performed for 10 min and mounted with Vectashield mounting medium (Vector Labs, CA). Stained tissues were imaged using a LSM 510 meta confocal microscope and images were processed using Photoshop CS6 software.

### Lineage tracing studies

For lineage tracing, we used the *αMHC:CreERT2* mouse strain and crossed the mice with *Rosa26-SmoM2/YFP* mice. To lineage label the pre-existing cardiomyocytes, we injected 4-hydroxytamoxifen in neonates at P0, P3, and P6 prior to MI. Following TM-injection, all the cardiomyocytes express EGFP and were labeled green. Control and *SmoM2-YFP*^*fl/+*^ mice received intraperitoneal injections of EdU (25 µg/g) and sacrificed at the time periods described. Immunohistochemical techniques were performed to detect the lineage-labeled cells as described above.

### Mouse ventricular cardiomyocyte isolation

Ventricular cardiomyocytes were isolated using previously published protocols^55^. Briefly, ventricles were dissected from P1 pups, minced in CBFHH (calcium and bicarbonate-free Hanks with Hepes) buffer. Subsequently, the minced ventricles were digested in CBFHH buffer containing 1.75 mg/ml of trypsin and 20 µg/ml of DNaseII (Sigma-Aldrich). Cells were preplated for 1 h (3 times) onto 100-mm primaria (Corning Life Sciences) dishes in culture medium containing 10% serum to remove fibroblasts. Unattached cardiomyocytes were plated at a desired density. Using this protocol, we routinely obtained >85–90% cardiomyocytes (confirmed using immunohistochemical techniques and an α-actinin antibody). After 12 h, the culture medium was changed and cells were subjected to the different treatments (SAG; 4 µg/ml and CyA; 5 µg/ml) and analyzed. For apoptotic pathway inhibition, neonatal cardiomyocytes were treated with SAG with or without the cell permeable small molecule inhibitor (Z-VAD-FMK; R&D Systems). For the EdU incorporation assay, cardiomyocytes were incubated with 20 µM EdU for 48 h and fixed using 4% PFA for 10 min at room temperature. P7 cardiomyocytes were isolated using a similar protocol as that of the P1 cardiomyocyte isolation protocol with modifications including the perfusion based tissue digestion using collagenase type 2 enzyme solution. We consistently obtained >95% rod-shaped binucleated cardiomyocytes using this protocol. For qPCR and FACS analysis, cells were harvested using trypsin and processed for further analysis. For the time-lapse microscopic experiments, isolated P7 mCherry + cardiomyocytes were plated on a glass-bottom Petri dish coated with fibronectin. After 2 h of attachment, the medium was changed (containing DMSO or SAG) and the Petri dish was placed in the environmental chamber for imaging. Adult cardiomyocyte isolation was performed as described previously^53^. Briefly, the adult heart tissue was dissociated by perfusion of a collagenase type 2 enzyme solution. Isolated cardiomyocytes were cultured in 1% serum medium in the presence of DMSO or SAG. For the EdU incorporation assay, cardiomyocytes were incubated with 20 µM EdU for 48 h and fixed using 4% PFA for 10 min at room temperature.

### Synthesis and Transfection of *Mycn* and *Gfp mRNAs*

PCR products with the T7 promoter site in the 5′ end for Mycn (Primers: Mycn T7 forward: TAATACGACTCACTATAGGGCACCATGCCCAGCTGCACCGCGTC, Mycn reverse: TTAGCAAGTCCGAGCGTGTTCGAT) and GFP (Primers: GFP T7 forward: AATACGACTCACTATAGGGCACCATGAGCGGGGGCGAGGAGCTG, GFP reverse: TTATCTGAGTCCGGACCTGTACAG), coding sequences were amplified from the respective plasmids (Mycn:Origene; MR207382L2, Gfp:Origene;TR30023). PCR products were purified and 500 ng of the PCR template was used for the in vitro synthesis and capped using the mMESSAGE mMACHINE T7 Ultra Kit (ThermoFisher # AM1345). The capped transcription reaction was performed at 37 °C for 14 h followed by the poly(A) tailing reaction. RNA was recovered using the mirVana miRNA isolation kit (ThermoFisher # AM1560). 1.5 μg of the purified RNA was used for the transfection experiment using isolated adult cardiomyocytes.

### Lentiviral constructs and infection

Lentiviral particles to overexpress Gli1 (Origene; MR227023L2), Mycn (Origenes; MR207382L2) were generated using standard protocols^56^. To knockdown Gli1 and Mycn, we obtained four unique 29-mer shRNA for Gli1 (Origene; TL500820) and Mycn (Origene; TL514180) and tested each of them using transient transfection assays in C2C12 myoblasts. The most efficient constructs were used to generate lentiviruses using standard protocols^56^. Cultured cardiomyocytes were infected with lentiviruses using Lentiblast reagent (OZBiosciences) as per the manufacturer′s instruction. After 12 h of infection, cells were washed twice using pre-warmed culture medium and EdU (20 µM) was added for an additional 36 h time period. Cells were fixed using 4% PFA for 10 min at room temperature and processed as described above using immunocytochemistry and qPCR techniques.

### hiPSC differentiation

The human iPSCs were commercially obtained from iPierian, Inc. The hiPSC differentiation protocol was adapted from the protocol described by Zhang et al. with slight modifications^57^. Briefly, cultured hiPSCs were plated on matrigel containing RPMI/B27 minus insulin, Actinin A, and CHIR-99021 for one day. The medium was then changed with RPMI/B27 minus insulin and BMP4, and FGF from d1 to d2. On d3, the medium was changed with RPMI/B27 minus insulin and IWP-4. The differentiated cells were maintained in RPMI/B27 with insulin medium from d5 to d60. Beating cardiomyocytes were observed by day 10 of differentiation. For HH signaling activation and inhibition, the differentiation medium was changed containing SAG and CyA together with EdU (20 µM) for 48 h. Immunohistochemical analysis was performed as described previously^58^.

### Bootstrap-based gene set analysis

The microarray dataset consisting of 18,560 genes and 9 time points (2, 6, 24, 48 h, 4 days, 7 days, 14 days, 21 days, and 35 days) from the regenerating newt heart following injury was downloaded from Newt-Omics (http://newt-omics.mpi-bn.mpg.de). For each gene, the expression levels across all *M* time points were scaled to a mean of zero and standard deviation of one. To examine whether a gene set *S* with the size of |*S*| is dynamically expressed postinjury, we computed the Euclidean distance between the mean expression profile of genes in set *S*, and the background expression profile, that is, the mean expression of all genes, as *d*. To generate a null distribution for *d*, we also computed the distance between the mean expression profile of randomly sampled |*S*| genes and the background expression profile, and repeated 1000 times. The bootstrapped distribution of the distance to background expression profile was represented as *d*^0^. Thus, the *p*-value was computed as:

$$\frac{{\mathop {\sum }\nolimits_{n = 1}^{1000} H\left( {d_{\boldsymbol{n}}^0\,\,> \,\,d} \right) + 1}}{{1000 + 1}}$$

where *H*(*x*) = 1, if *x* > 0, otherwise 0. We reported the significantly changed gene sets with an adjusted *p*-value < 0.05.

### ChIP-PCR assay

The ChIP-PCR assay for endogenous Gli1 was performed as previously described^19^. Briefly, the cultured neonatal cardiomyocytes were harvested in lysis-buffer and the DNA-protein complex was immunoprecipitated using biotinylated anti-Gli1 antibody (R&D Systems), followed by streptavidin-conjugated magnetic beads. PCR was performed to detect the target region using the following primers; Fwd: 5′-CTTCGCAAGTACCGCTTC-3′; Rev: 5′-ATATCCCCCGAGCTTCAA-3′.

### Statistical analysis

Statistical significance was determined using the Student’s *t*-test and one-way ANOVA (non-parametric) test and a *p*-value < 0.05 was considered a statistically significant change.

Additional details regarding experimental procedures can be found in the Supplementary Information section.

## Electronic supplementary material

Supplementary Information

Description of Additional Supplementary Files

Supplementary Movie 1

Supplementary Movie 2

## Data Availability

All data will be made available by the corresponding author upon reasonable request.
