# Supplementary material for: A conserved HH-Gli1-Mycn network regulates heart regeneration from newt to human
Source: Nat Commun. 2018 Oct 12;9:4237. doi: 10.1038/s41467-018-06617-z (PMC6185975; doi:10.1038/s41467-018-06617-z)
Supplement: Supplementary file 1 — Supplementary Information [file 41467_2018_6617_MOESM1_ESM.pdf]

A conserved HH-Gli1-Mycn network regulates heart regeneration from newt to human

Singh et al;

Supplementary Fig 1

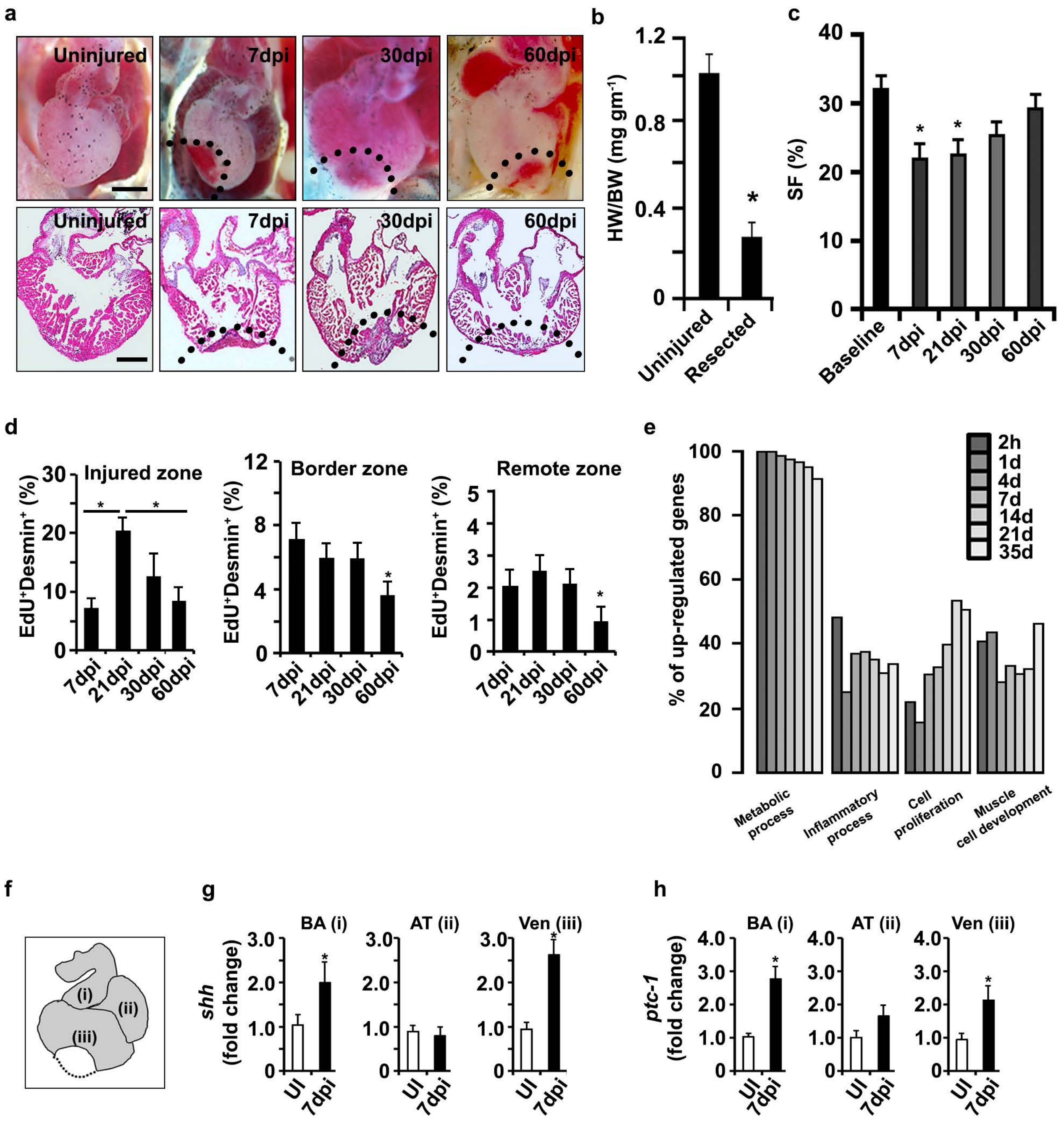

Supplementary Fig. 1 (related to Fig. 1). Resection injury results in a robust proliferative response and increased expression of HH signals in the regenerating heart. (a) Representative whole mount image analysis (top panels) and histological examination (bottom panels) of the regenerating newt heart (injured region designated with a dotted line) at each time period following injury. **(b)** HW/BW quantification of the uninjured and the resected portion of the heart (n=10). Note, approximately 25% of the heart was resected. **(c)** Echocardiographic measurement (SF) of the regenerating newt heart at 7dpi, 21dpi, 30dpi and 60dpi (n=5 for each time period). **(d)** Quantitative analysis of Desmin$^+$-EdU$^+$ cardiomyocytes in the injured region of the regenerating newt heart. Quantitative analysis represents counts from four randomly selected fields at 20X magnification from four replicates. **(e)** Bar graph showing the dysregulated biological processes including metabolic processes, cell migration, cardiac muscle development and cellular proliferation in the regenerating newt heart. **(f-h)** Schematic outlining the newt heart anatomy (f) and qPCR analysis for *shh* (g) and *ptc-1* (h) transcripts from the bulbous arteriosus [BA (i)], atrium [AT (ii)] and ventricle [ven (iii)] from the regenerating newt heart. Data represent mean $\pm$ SEM (*p < 0.05) and scale bars = 500 μm (Panel a, top) and 100 μm (Panel a, bottom).

Supplementary fig 2

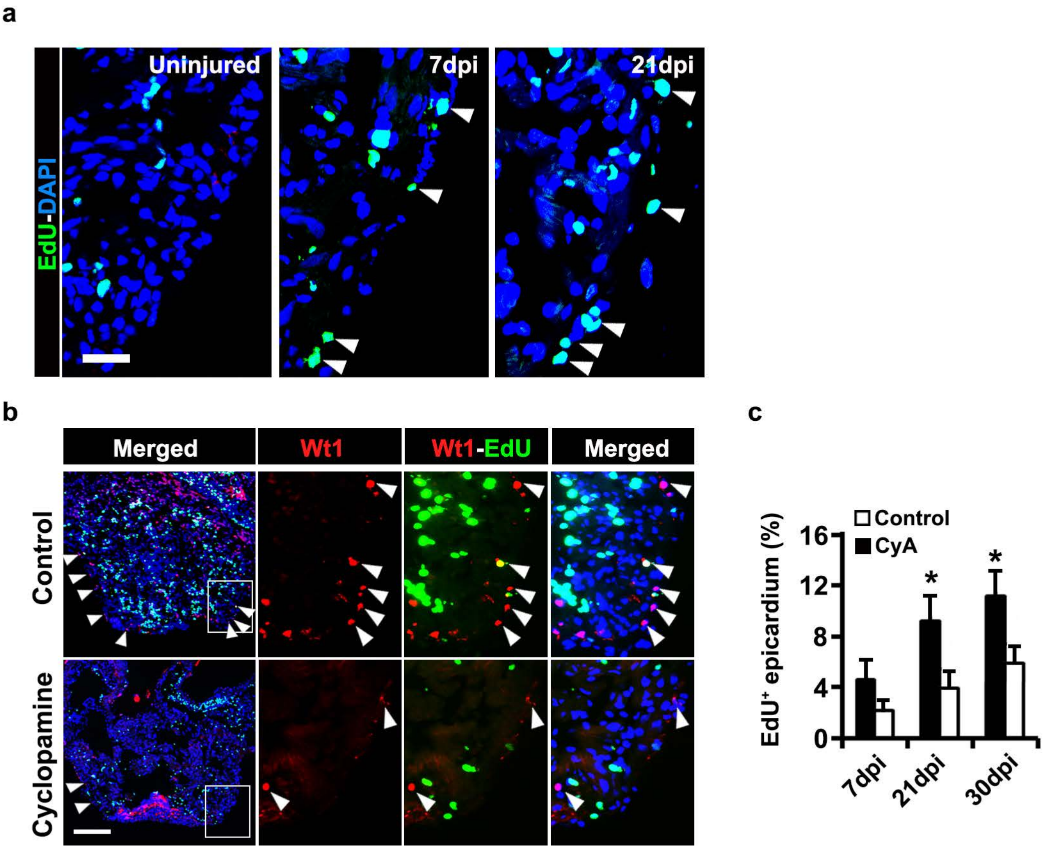

Supplementary Fig. 2 (related to Fig. 1). Inhibition of HH signaling reduces epicardial cell proliferation following apical resection injury. (a) Immunohistochemical analysis of the EdU^{+} cells in the regenerating newt heart from uninjured, 7dpi and 21dpi heart tissue. The white arrowheads indicate the EdU^{+} cells in the outermost layer of the section. **(b, c)** Immunohistochemical staining (b) and quantification (c) of Wt1^{+}- EdU^{+} epicardial cells in the regenerating heart from control and CyA-treated newts at the designated time periods following injury (n=4). White arrowheads indicate the Wt1^{+}- epicardial cells following injury. Quantitative analysis represents counts from four different fields at 20X magnification from four replicates. Data represent mean $\pm$ SEM (*$p < 0.05$) and scale bars = 100 μm (Panel a) and 50 μm (Panel b).

Supplementary Fig 3

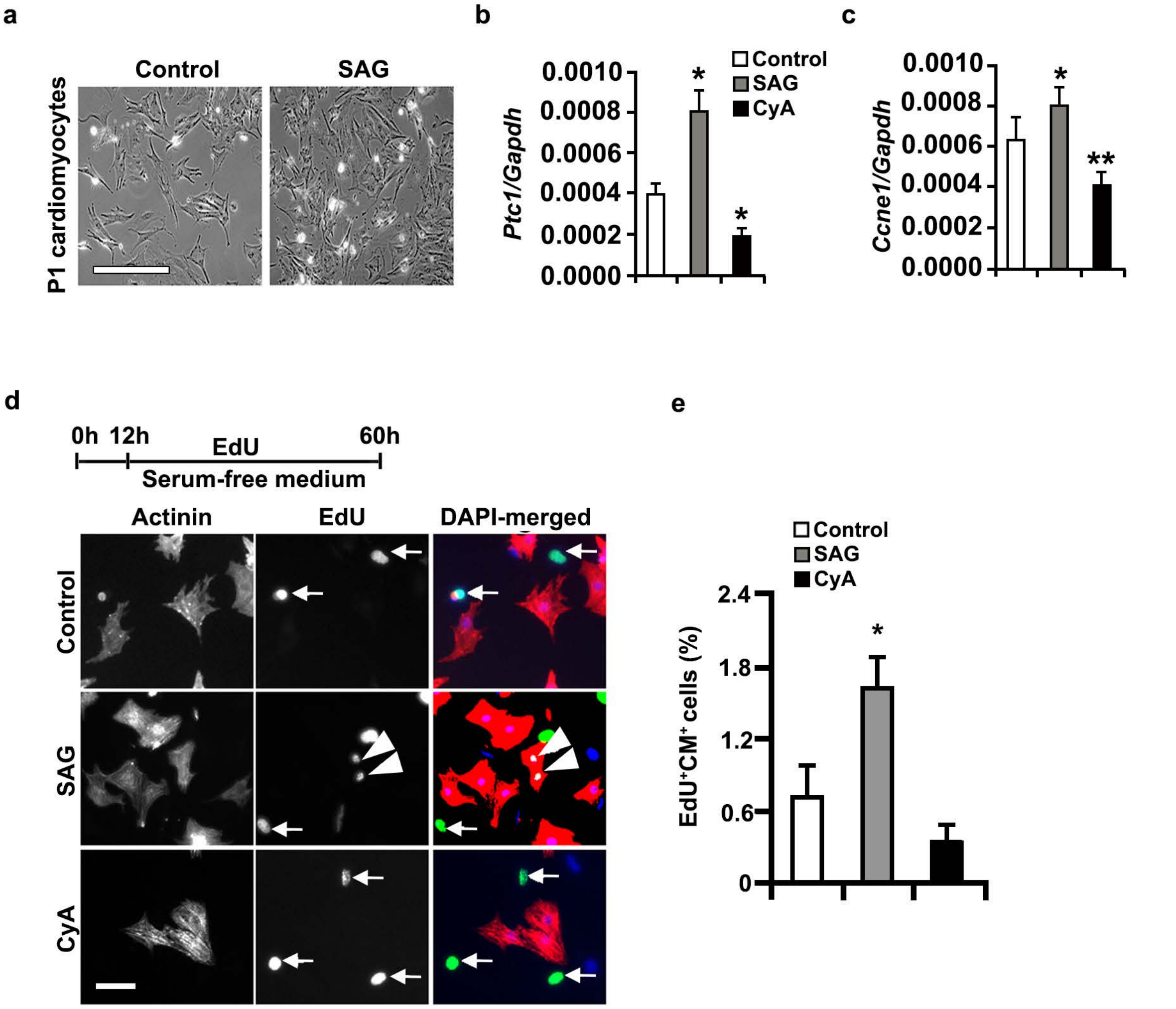

Supplementary Fig. 3 (related to Fig. 2). HH signaling regulates mouse neonatal cardiomyocyte proliferation. (a) Representative phase contrast image of control and SAG treated P1 cardiomyocytes. **(b, c)** qPCR analysis for *Ptc1* and *Ccne1* transcripts from control (white bar), SAG (grey bars) and CyA (black bars) treated neonatal cardiomyocytes (n=3 for each group). **(d, e)** Immunohistochemical (d) and quantitative analysis (e) of α-Actinin^{+}-EdU^{+} cardiomyocytes from control (white bar) SAG (grey bar) and CyA (black bar)-treated isolated neonatal cardiomyocytes in serum-free conditions (n=3 for each group). Arrowheads indicate EdU^{+}-cardiomyocyte nuclei and arrows indicate EdU^{+}-nonmyocyte nuclei. Nuclei were stained with DAPI. Data in panels b, c and e represent mean $\pm$ SEM (*p < 0.05) and scale bars = 200 μm.

Supplementary Fig 4

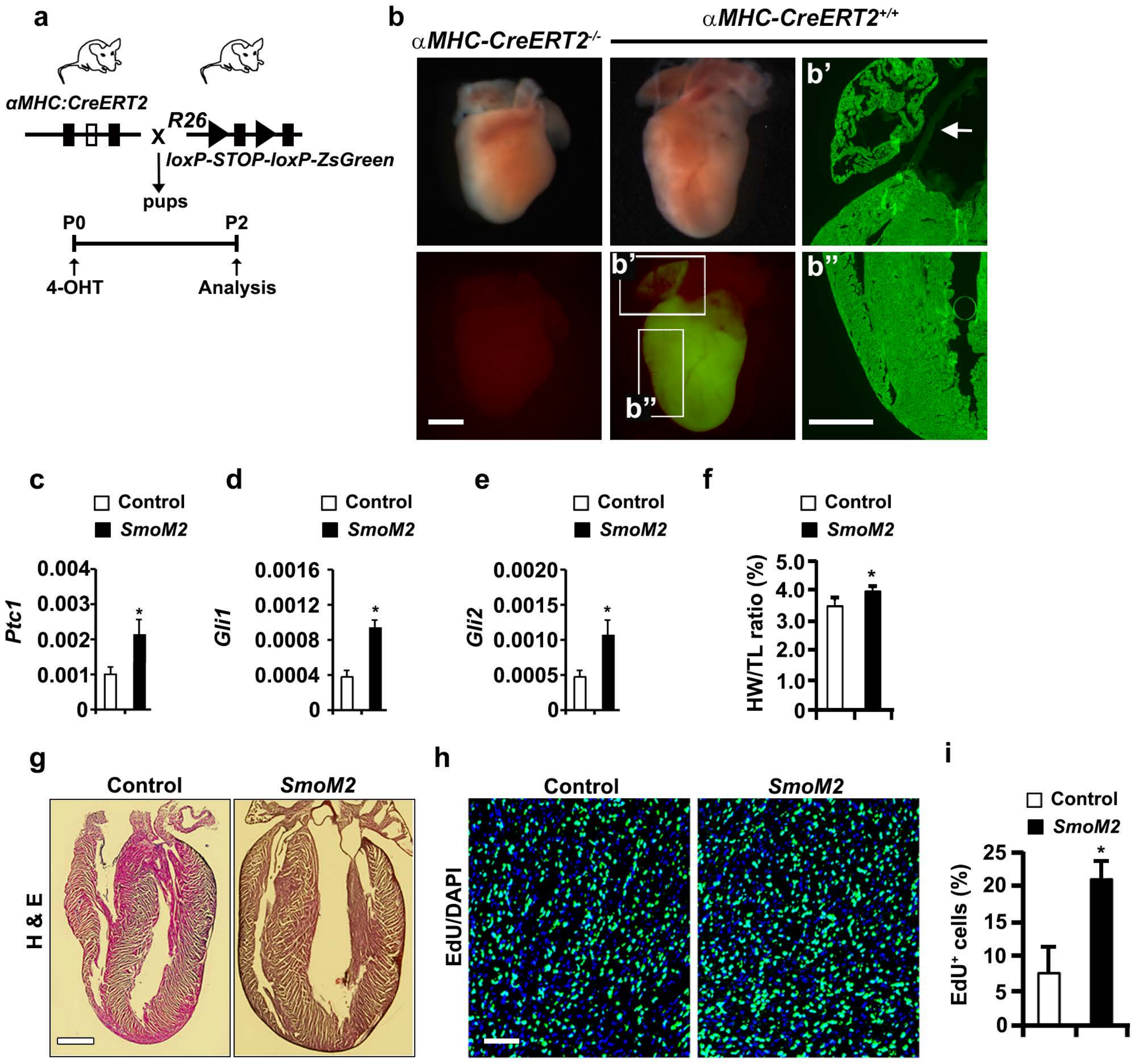

Supplementary Fig. 4 (related to Fig. 3). Cardiac-specific modulation of HH signaling regulates cardiomyocyte proliferation *in vivo*. (a) Schematic outlining the experimental protocol to examine the specificity of inducible Cre expression within cardiomyocytes in the postnatal heart. **(b)** Representative whole mount and histological analysis of *ZsGreen* reporter from control and 4-hydroxy tamoxifen (80µg/gm) injected mice obtained by crossing *αMHC:CreERT2* with *Rosa26-ZsGreen* mice at P2. The boxed region is magnified in panel b' and b''. Note the presence of green fluorescence protein in myocardium (cardiomyocytes) and absence of GFP fluorescence in the noncardiomyocytes (arrow) (i.e. great vessels such as the aorta). **(c-e)** qPCR analysis of *Ptc1, Gli1,* and *Gli2* transcripts using RNA isolated from control and *SmoM2* heart tissue at P7 (n=3 from each group). **(f)** Quantitative assessment of heart weight and tibia length (HW/TL) ratio of the hearts obtained from control and *SmoM2* pups (n=5 for each group). **(g)** Representative H&E staining of the hearts obtained from control and *SmoM2* pups (n=5 for each group). **(h, i)** EdU staining (h) and quantification (i) of the EdU^+ cells from control and *SmoM2* hearts. Quantitative analysis represents counting of three randomly selected fields from three biological replicates. Data from panels c, d, e, f and i represent mean $\pm$ SEM (*$p < 0.05$) and scale bars = 200 µm (Panels b', b'', b") and 100 µm (Panels g, h).

Supplementary Fig 5

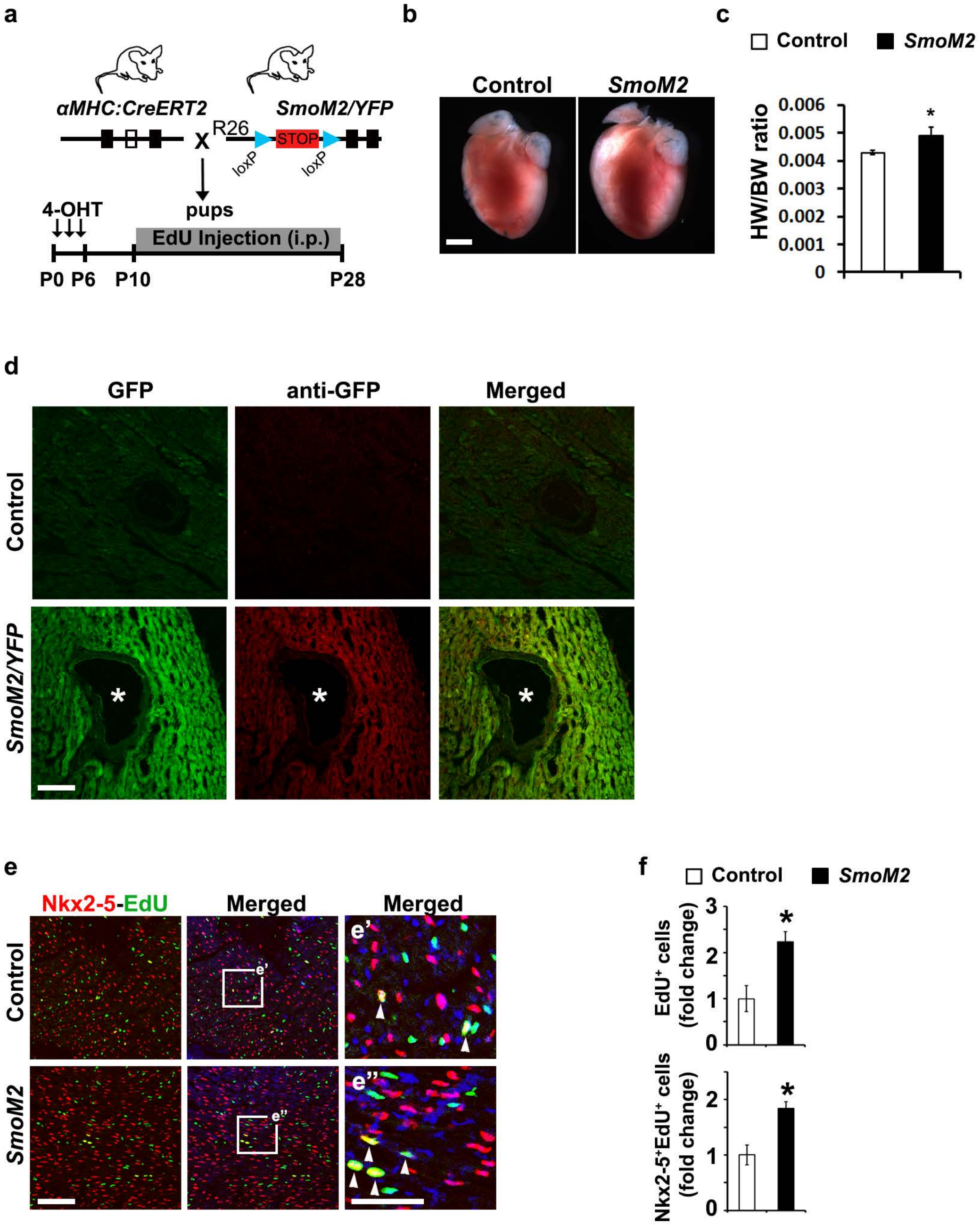

Supplementary Fig. 5. Induction of HH signaling promotes *in vivo* cardiomyocyte proliferation in the late juvenile stage of the mouse. (a) Schematic for the experimental protocol to study the activation of HH signaling and EdU labeling in the postnatal heart. EdU (shaded area) was injected intraperitoneally (i.p.) every three days. **(b)** Representative wholemount heart images obtained from control and *SmoM2* pups at P28. **(c)** Quantitative assessment (HW/BW ratio) of the hearts obtained from control and *SmoM2* mice at P28 (n=4 in each group). **(d)** Representative immunostained images showing the expression of the fluorescent reporter at P28. The asterisk designates the lumen of a muscular artery within the ventricular tissue. Note the absence of GFP expression and staining in the vascular wall. **(e, f)** Immunostaining (e) and quantification (f) of EdU^+ cells (top) as well as $Nkx2\text{-}5^+$-EdU^+ cardiomyocytes (bottom) in control and *SmoM2* hearts. The boxed region is magnified in panel e' and e''. The white arrowheads indicate the cardiomyocytes that had undergone a proliferative event within the myocardium. Data from panels c and f represent mean $\pm$ SEM (*$p < 0.05$) and scale bars = 500 μm (Panels b) and 100 μm (Panels d, e).

Supplementary Fig 6

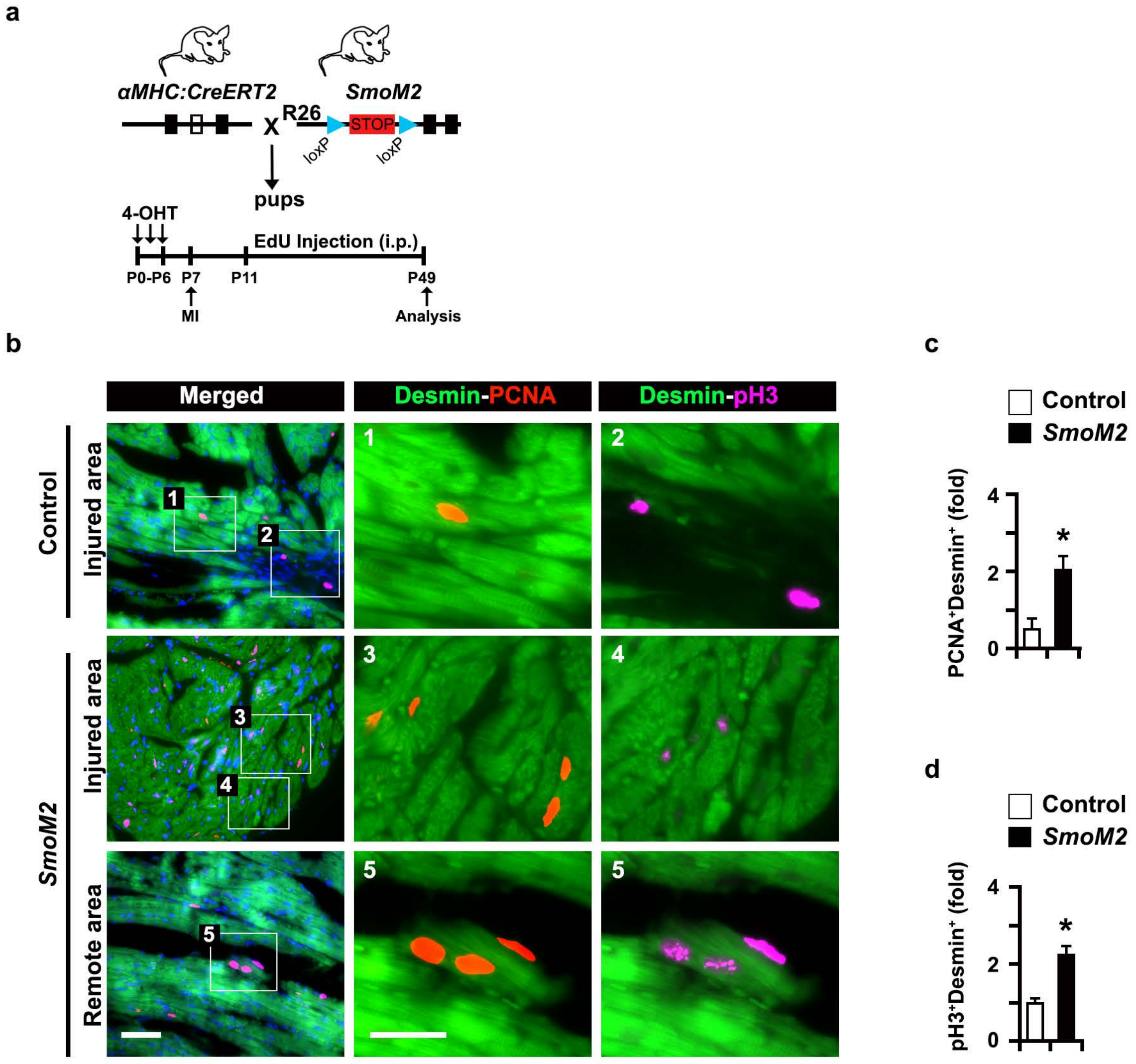

Supplementary Fig. 6 (related to Fig. 6). Induction of HH signaling pathway results in enhanced cardiomyocyte proliferation following injury. (a) Schematic outlining the experimental design for Smo activation and cardiac regeneration analysis following myocardial injury. **(b)** Immunostaining of $Desmin^+$-$PCNA^+$ cardiomyocytes and $Desmin^+$-$pH3^+$ cardiomyocytes in control and *SmoM2* hearts at 42 days post-MI. Boxed regions in panel "B" are magnified in panels 1, 2, 3, 4, and 5. **(c, d)** Quantitative analysis of $Desmin^+$-$PCNA^+$ (c) and $Desmin^+$-$pH3^+$ cardiomyocytes (d) in control and *SmoM2* hearts. Quantitation represents counts from four different fields at 20X magnification from three replicates. Data in panels c and d represent mean $\pm$ SEM (*$p < 0.05$) and scale bars = 100 μm.

Supplementary Fig 7

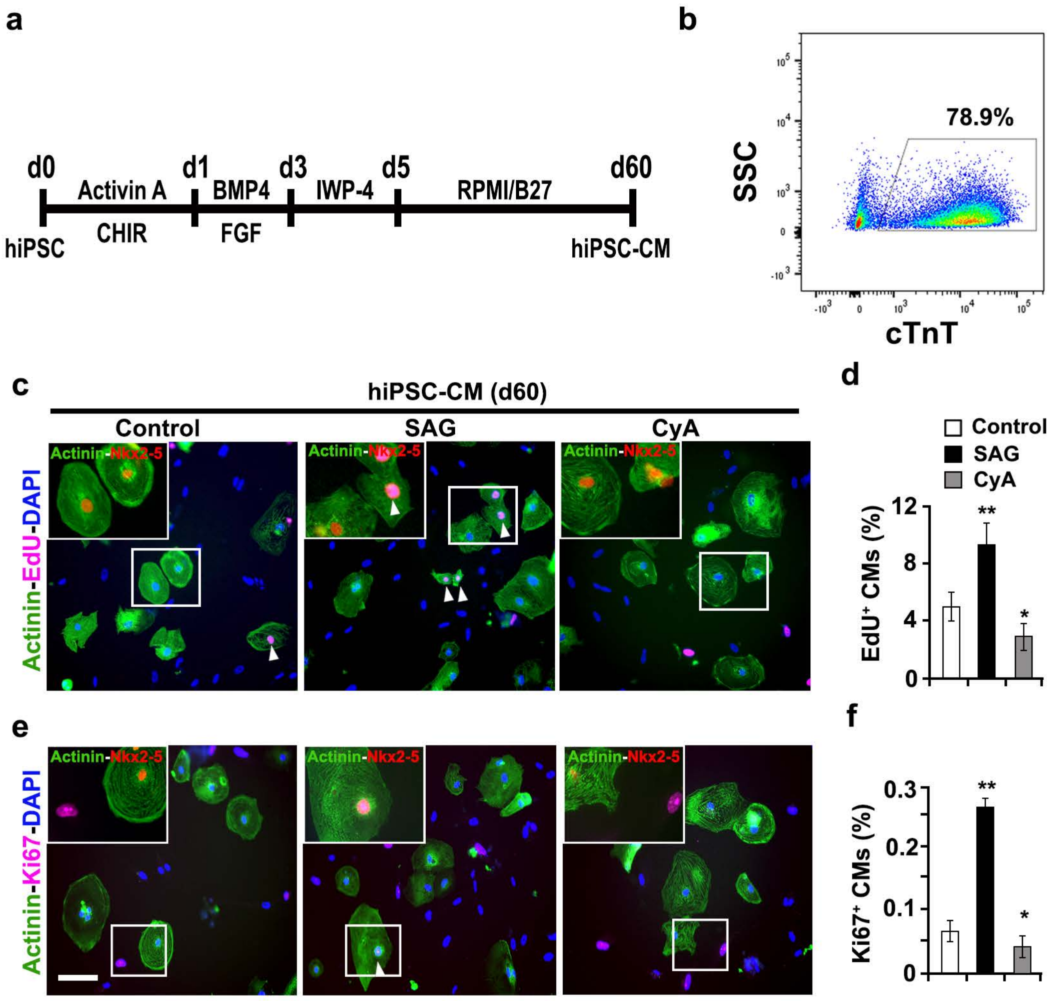

Supplementary Fig. 7. Induction of HH signals promotes proliferation of d60 hiPSC-CMs. (a) Schematic of hiPSC-derived cardiomyocyte differentiation protocol as previously described[1]. **(b)** Representative FACS analysis for intracellular cTnT using hiPSC-CMs. **(c-f)** Immunohistochemical (c, e) and quantification (d, f) of α-Actinin^{+}-EdU^{+} and α-Actinin^{+}-Ki67^{+} d60 hiPSC-CMs from control, SAG and CyA treated conditions. The boxed region is further magnified and shown in the left corner of the images. Quantitative analysis represents the counting of four randomly chosen fields from three replicates (n=1935 cardiomyocytes for each condition). Data in panels d and f represent mean ± SEM (*$p < 0.05$; **$p < 0.01$) and scale bars = 200 μm.

Supplementary Fig 8

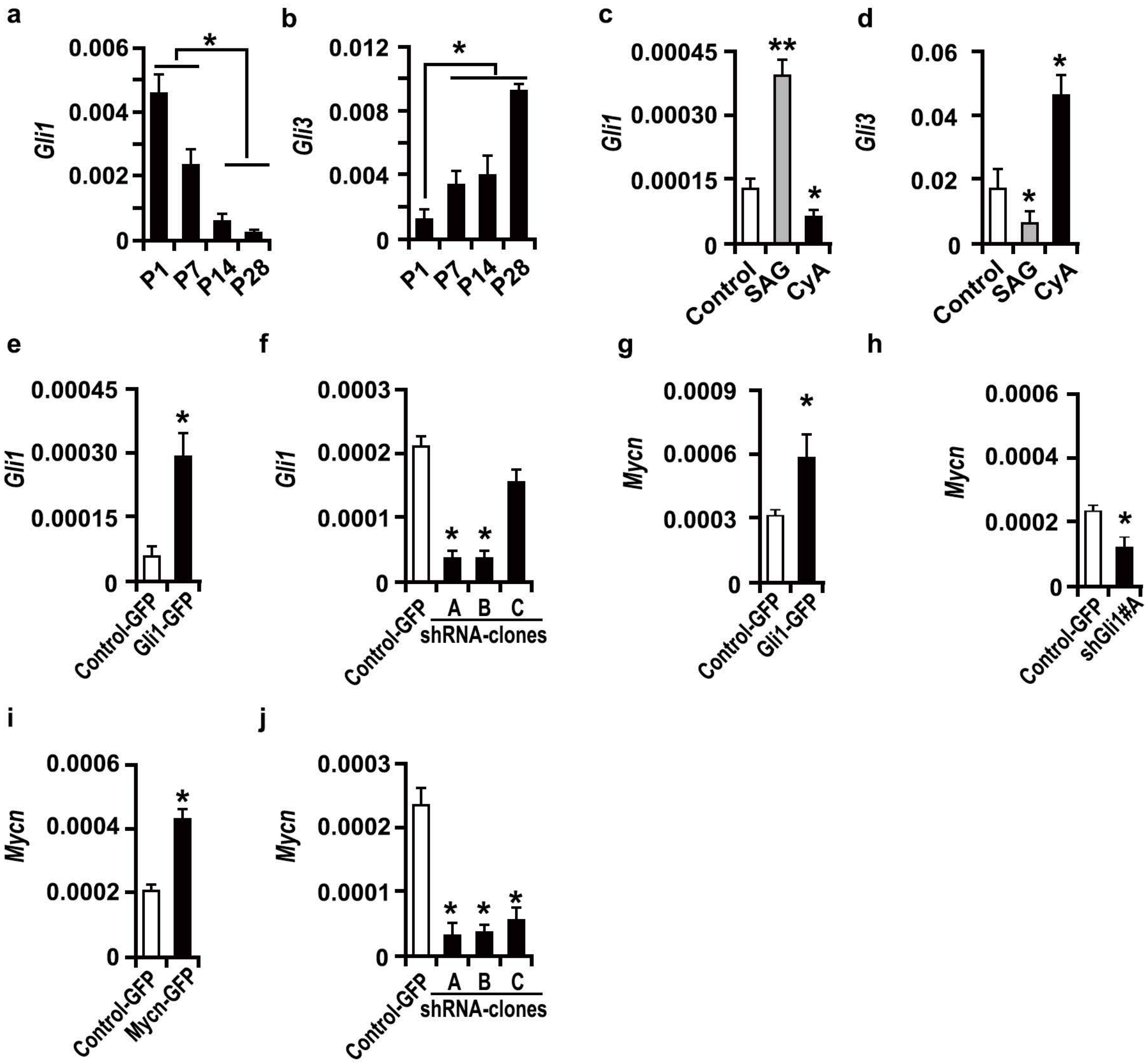

Supplementary Fig. 8 (related to Fig. 8). Gli1-Mycn network regulates neonatal cardiomyocyte proliferation. **(a, b)** qPCR analysis of *Gli1* and *Gli3* transcripts using RNA isolated from P1-P28 wild type heart tissue (n=3 at each time period). **(c, d)** qPCR analysis of *Gli1* and *Gli3* transcripts using RNA isolated from control, SAG and CyA-treated P1 cultured cardiomyocytes (n=3 for each group). **(e, f)** qPCR analysis of *Gli1* transcripts following lentiviral Gli1 overexpression or following Gli1 knockdown using three different shRNA clones. **(g, h)** qPCR analysis of *Mycn* transcripts following Gli1 overexpression and Gli1 knockdown (using shRNA clone B) in P1 cultured cardiomyocytes. **(i, j)** qPCR analysis of *Mycn* transcripts following lentiviral Mycn overexpression or following Mycn knockdown using three different shRNA clones. Data in panels a-j represent mean ± SEM (*$p < 0.05$; **$p < 0.01$).

Supplementary Fig 9

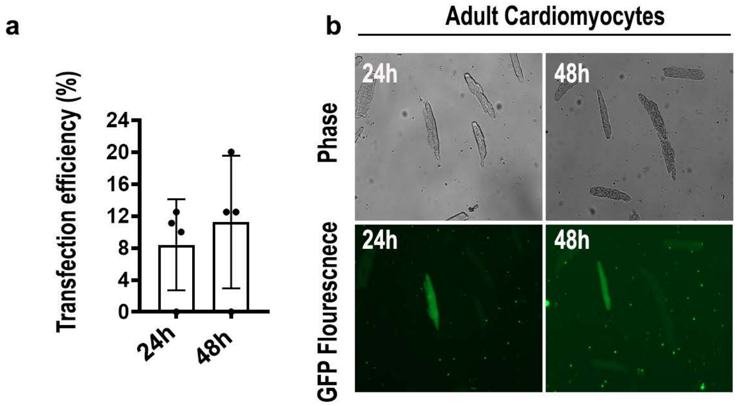

Supplementary Fig. 9 (related to Fig. 9). mRNA mediated transfection of *Gfp* and *Mycn* in adult cardiomyocytes. **(a, b)** Quantification (a) and microscopic images (b) of the Gfp transfected adult cardiomyocytes at 24h and 48h post-transfection.

Supplementary table 1: Gli1 ChIPseq data analysis based on binding motif proximity, heart expression and their functions.

refseq genome	entrezgene	is.cell.cycle	proli	dist score	Ht exp	HH target	Total Score
NM_080554	Mycn	TRUE	1	0.356	1	1	3.356
NM_001042653	Id3	TRUE	1	0.350	1	1	3.350
NM_007836	Hexim1	TRUE	1	0.328	1	1	3.328
NM_023873	Rara	TRUE	1	0.272	1	1	3.272
NM_008957	Gipc1	TRUE	1	0.132	1	1	3.132
NM_008957	Htra2	TRUE	1	0.076	1	1	3.076
NM_178884	Dicer1	TRUE	1	0.02	1	1	3.02
NM_009832	Psmd11	TRUE	1	1	0	1	3
NM_008321	Ensa	TRUE	1	0.972	0	1	2.972
NM_010817	Dhcr24	TRUE	1	0.468	0.5	1	2.968
NM_008709	Ccnk	TRUE	1	0.944	0.5	0.5	2.944
NM_138753	Wdr6	TRUE	1	0.916	0	1	2.916
NM_009024	Hjurp	TRUE	1	0.888	0	1	2.888
NM_018771	Hjurp	TRUE	1	0.86	0	1	2.86
NM_019752	Tpd52l1	TRUE	1	0.832	0	1	2.832
NM_148948	Oip5	TRUE	1	0.804	0	1	2.804
NM_178616	Oip5	TRUE	1	0.776	0	1	2.776
NM_001026212	Oip5	TRUE	1	0.748	0	1	2.748
NM_053272	Dynll1	TRUE	1	0.72	0	1	2.72
NM_008957	Psmd7	TRUE	1	0.216	0.5	1	2.716
NM_031392	Smpd3	TRUE	1	0.692	0	1	2.692
NM_008957	Pds5b	TRUE	1	0.188	0.5	1	2.688
NM_172505	Ptch1	TRUE	1	0.664	1	0	2.664
NM_172505	Psmd5	TRUE	1	0.636	0	1	2.636
NM_009413	Gadd45a	TRUE	1	0.608	0	1	2.608
NM_001042653	Ptch1	TRUE	1	0.58	1	0	2.58
NM_001042653	Cep70	TRUE	1	0.524	0	1	2.524
NM_019682	Ptch1	TRUE	1	0.496	1	0	2.496
NM_021491	Ptch1	TRUE	1	0.44	1	0	2.44
NM_175310	Ptch1	TRUE	1	0.412	1	0	2.412
NM_008957	Ptch1	TRUE	1	0.384	1	0	2.384
NM_008957	Ptch1	TRUE	1	0.3	1	0	2.3
NM_008957	Ptch1	TRUE	1	0.244	1	0	2.244
NM_008957	Ptch1	TRUE	1	0.16	1	0	2.16
NM_008957	Dusp3	TRUE	1	0.104	0	1	2.104
NM_028207	Obsl1	TRUE	1	0.048	0	1	2.048

Supplementary Table 1 (related to Fig. 8). ChIPseq analysis of Gli1 downstream targets. Gli1 ChIPseq data analysis based on binding motif proximity, heart expression and their functional role.

Supplementary Movie-1 (related to Fig. 4). Time lapse microscopy of culture αMHC-mCherry^{+} P7 cardiomyocytes treated with DMSO.

Supplementary Movie-2 (related to Fig. 4). Time lapse microscopy of culture αMHC-mCherry^{+} P7 cardiomyocytes treated with SAG.

Supplementary reference

1 Dubois, N. C. *et al.* SIRPA is a specific cell-surface marker for isolating cardiomyocytes derived from human pluripotent stem cells. *Nat Biotechnol* **29**, 1011-1018, doi:10.1038/nbt.2005 (2011).
