# Supplementary material for: A conserved HH-Gli1-Mycn network regulates heart regeneration from newt to human
Source: Nat Commun. 2018 Oct 12;9:4237. doi: 10.1038/s41467-018-06617-z (PMC6185975; doi:10.1038/s41467-018-06617-z)
Supplement: Supplementary file 2 — Description of Additional Supplementary Files [file 41467_2018_6617_MOESM2_ESM.pdf]

Description of Additional Supplementary Files

**File Name:** Supplementary Movie 1
**Description:** Time lapse microscopy of cultured αMHC-mCherry+ P7 cardiomyocytes treated with DMSO (related to Fig. 4)

**File Name:** Supplementary Movie 2
**Description:** Time lapse microscopy of cultured αMHC-mCherry+ P7 cardiomyocytes treated with SAG (related to Fig. 4)
